# Supplementary material for: Genetic modifiers of response to thalidomide in transfusion-dependent beta-thalassemia patients: a whole-exome sequence analysis
Source: PeerJ. 2025 Oct 7;13:e20038. doi: 10.7717/peerj.20038 (PMC12513377; doi:10.7717/peerj.20038)
Supplement: Supplemental Information 1 [file peerj-13-20038-s001.docx]

**Supplementary file 1:** whole exome sequencing method

In this study, the Whole exome region was captured by probe hybridization method. Sequencing was performed with Next generation sequencing method.

**Library Preparation**

The DNA was fragmented using ultrasonicator. The interrupted DNA fragments were constructed into a high throughput sequencing library via following steps i.e. terminal repair, adding base A tail, adaptor ligation, purification and pre amplification exon capture and PCR enrichment.

The size and concentration of each sample was determined. Qubit fluorimeter was used for this purpose to measure concentration of DNA in each sample.

**Qc of sequencing of data**

**Sequencing Data Specification**

The optical signal under the fluorescent channels scanned by the built-in software RTA were converted to base-calling files in real-time. After the base-calling, Illumina's official software bcl2fastq (v2.17) was used to de-multiplex the data according to the sample index sequence and convert it into FASTQ format. The primary analysis procedure of the sequencer HCS software determines whether the reads would pass filter (the first 25 cycles has no more than 2 base which chastity value less than 0.6) based on the purity of the first 25 cycles of the read signal or not. The PF clusters stored in the FASTQ format after conversation, is called PF data or raw data. For paired-end data, sequence data consists of two FASTQ files that hold each end of the sequence read.

**Sequencing Data Filtering**

Using QC software cutadapt filters, the raw reads were processed to obtain clean data for further analysis as per following steps:

(1) Primer and adapter sequence were removed from reads.

(2) End bases of reads having quality value less than 20 were removed.

(3) Reads having ratio of N-base >10% were discarded.

(4) Pair-end reads with length < 75bp after trimming were discarded.

The adapter used for Illumina sequencing is as follows:

P7 adapter (Read1): AGATCGGAAGAGCACACGTCTGAACTCCAGTCAC

P5 adapter (Read2): AGATCGGAAGAGCGTCGTGTAGGGAAAGAGTGT.

**Statistics of Data Output and Sequencing Quality**

For all samples, the proportion of effective data was higher than 85%, Q20 was higher than 90%, Q30 was higher than 80% and GC content was about 50%.

**Mapping Sequences Against Reference Genome**

Sentieon software was used for sequence alignment with reference human genome hg19, sorting, marking duplication, quality correction and detection of mutation.

Samtools software was used to determine depth, coverage, mapping ratio and duplication rate .

**Statistics of sequencing depth, coverage and alignment**

**F**or qualified samples, the mapping rate was 95%. Mean target coverage was 90% and mean target depth was not lesss than 60x.

**Variants Detection**

Single nucleotide variants (SNV) and insertions-deletions (InDel) detection was performed using sentieon software. SNV annotation was performed using Annovar software regarding following four aspects of content:

(1) Annotating gene and region: this part includes the location information of the variation site, gene name, gene region of the site, variation type and whether it affects the translation of protein;

(2) Annotating the mutation information in database: this part includes the frequency and clinical information of mutation loci in avsnp147, 1000G, ExAc, Cosmic, and Clinvar.

(3) Predicting conservation and harmfulness: this part includes the prediction of the influence of the variation site on protein structure and function. The information is given regarding SIFT, Polyphen2, CADD and MutationTaster2.

(4) Other information: including the genotype of the mutation site, the depth of sequencing, the number of reads supporting the variation, etc.

**Detection of InDels**

Indels were obtained by sentieon, and the results were annotated by Annovar and attached.

**Statistics of InDels**

The distribution of region and types which indels occurred and effect on protein translation, and the number of homozygosity and heterozygosity were counted

**SNV Filtering**

Filtering in variant-level was done using default filtering criteria as follows:

1) Frequency filtering: Variants whose MAF > 0.01 in 1000Genomes and ExAC were excluded;

2) Non-Coding region filtering: Variants not in exonic or splicing region were excluded;

3) Synonymous variants were excluded.

4) Keeping deleterious Variants: Variants are kept if half of SIFT, Polyphen2, MutationTaster2 or CADD predict its deleterious effect.

**Results for InDel Filtering**

Filtering base on mutation: Default filtering criteria was as following:

1) Frequency filtering: Variants whose MAF > 0.01 in 1000Genomes and ExAC are excluded.

2) non-Coding region filtering: Variants not in exonic or splicing region are excluded.

3) Synonymous variants are excluded.
