# Supplementary material for: Genetic modifiers of response to thalidomide in transfusion-dependent beta-thalassemia patients: a whole-exome sequence analysis
Source: PeerJ. 2025 Oct 7;13:e20038. doi: 10.7717/peerj.20038 (PMC12513377; doi:10.7717/peerj.20038)
Supplement: Supplemental Information 2 [file peerj-13-20038-s002.docx]

**Supplementary file 2:** Quality control of exome data

Sample coding for figures is given in below table.

| **NR1** | Sample 6 |
| --- | --- |
| **NR2** | Sample 12 |
| **NR3** | Sample 15 |
| **NR4** | Sample 17 |
| **NR5** | Sample 20 |
| **NR6** | Sample 21 |
| **ER1** | Sample 3 |
| **ER2** | Sample 4 |
| **ER3** | Sample 5 |
| **ER4** | Sample 7 |
| **ER5** | Sample 8 |
| **ER6** | Sample 9 |
| **ER7** | Sample 10 |
| **ER8** | Sample 11 |
| **ER9** | Sample 13 |
| **ER10** | Sample 14 |
| **ER11** | Sample 16 |
| **ER12** | Sample 18 |
| **ER13** | Sample 19 |
| **ER14** | Sample 30 |

NR= non responder, ER=Excellent responder

**Sequencing Quality And Error Rate**


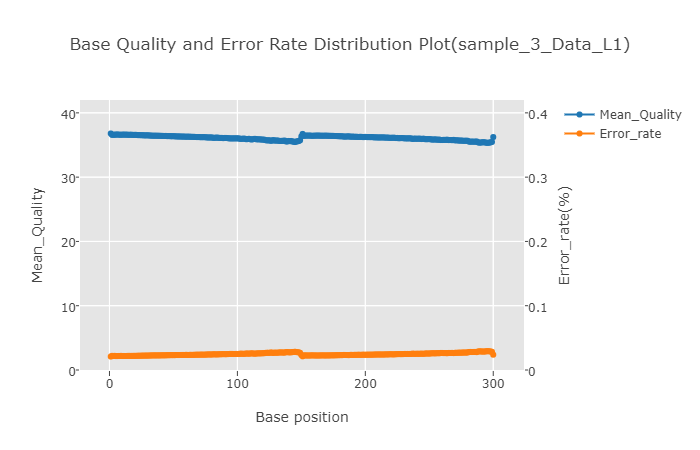

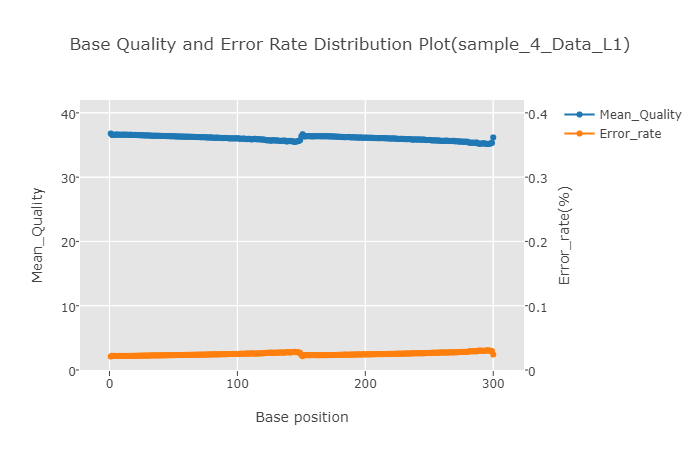


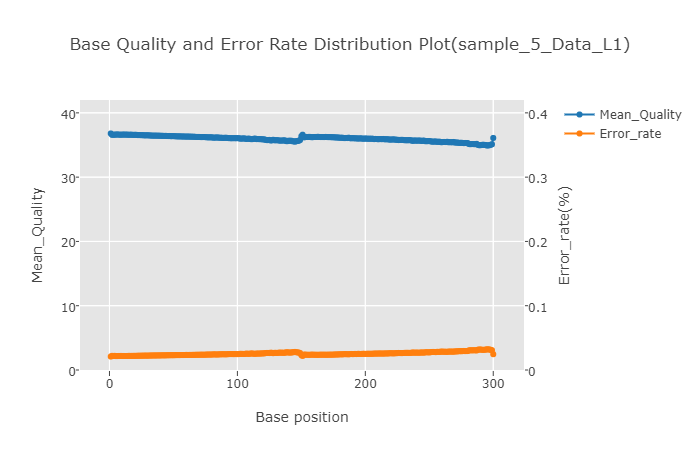

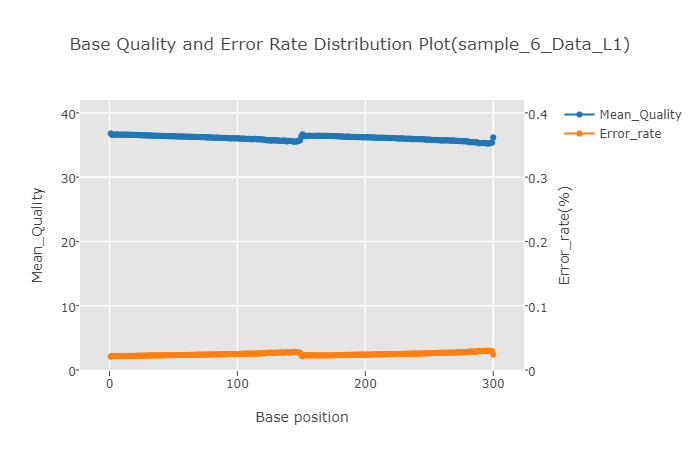


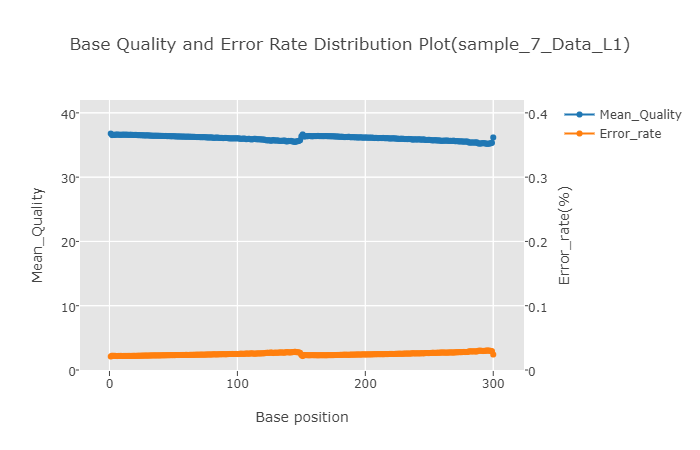

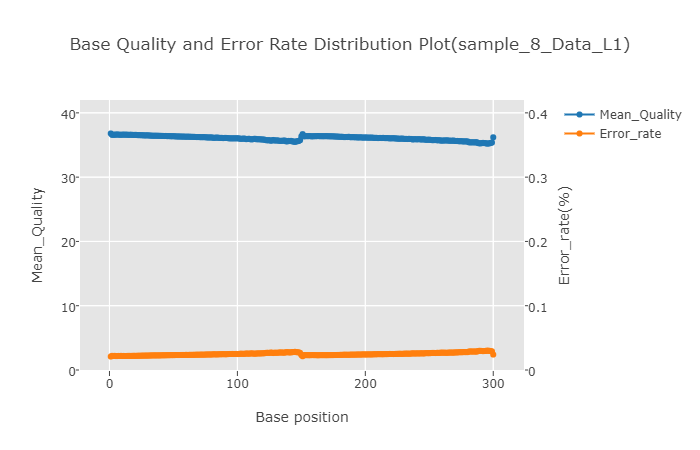

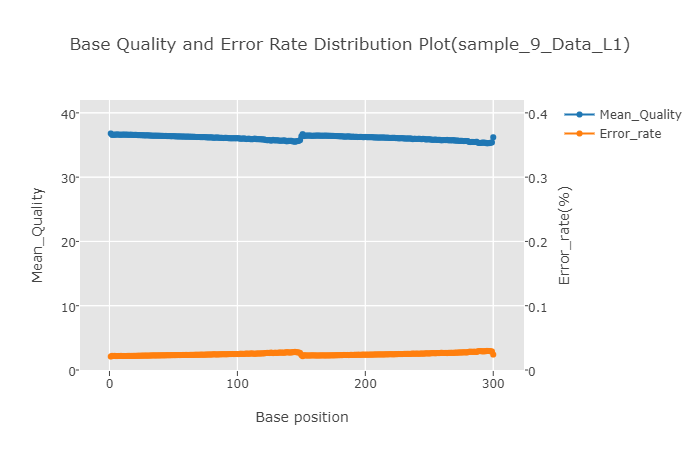

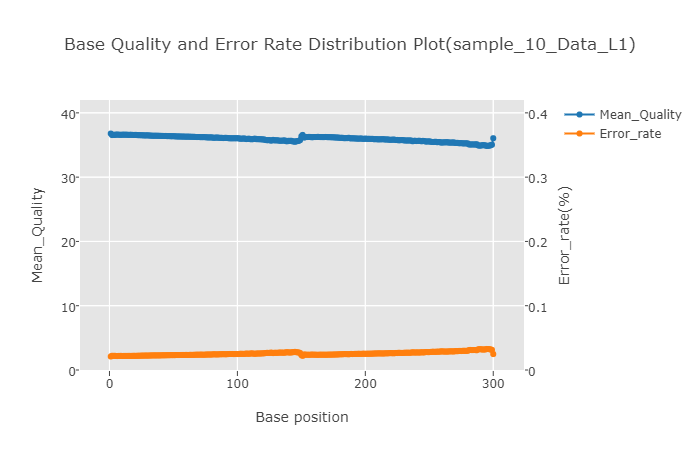


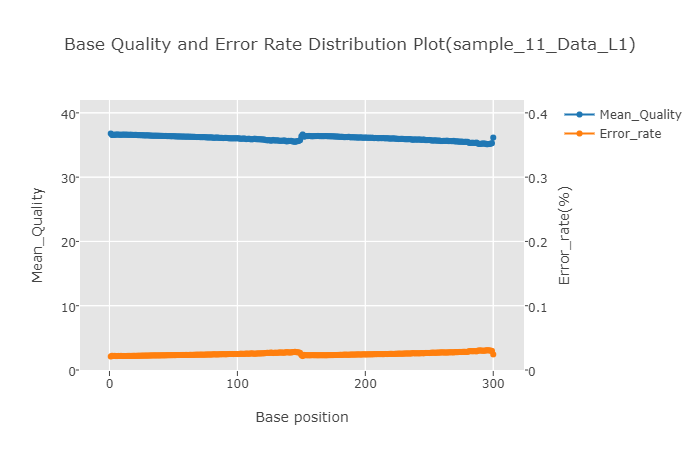

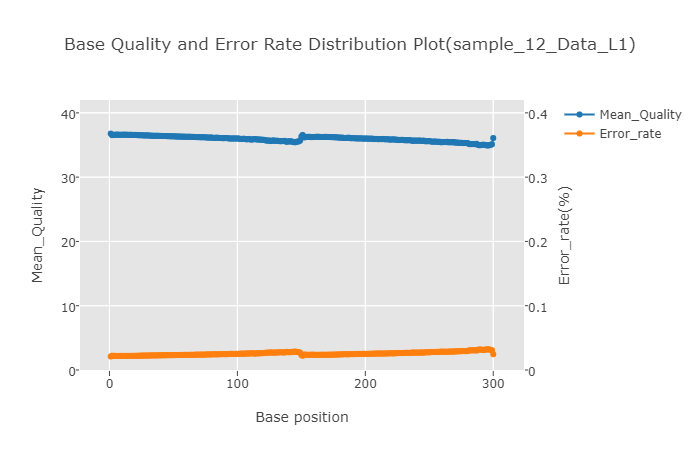


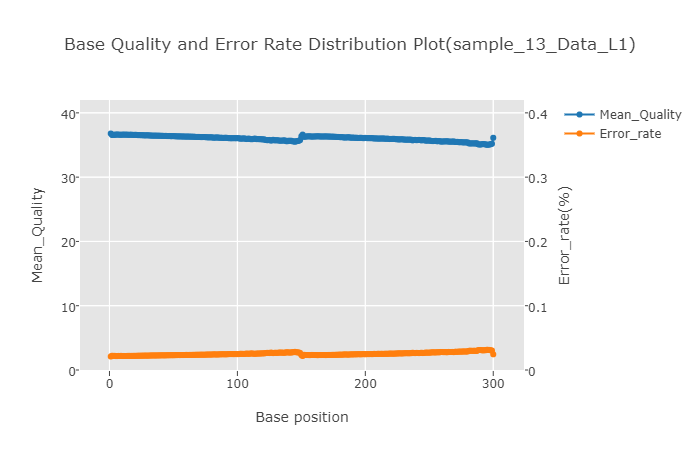

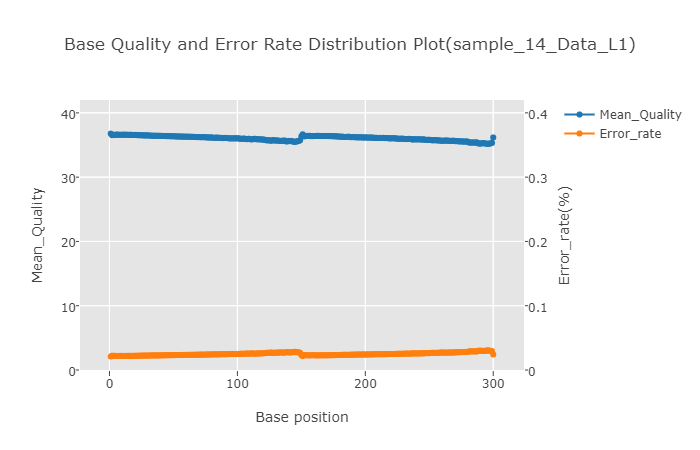


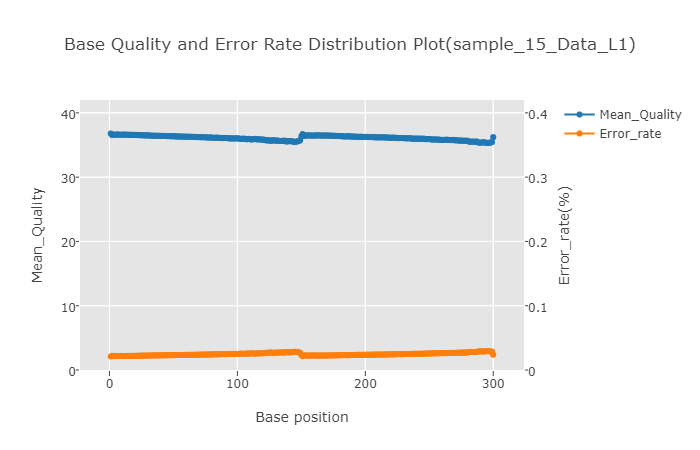

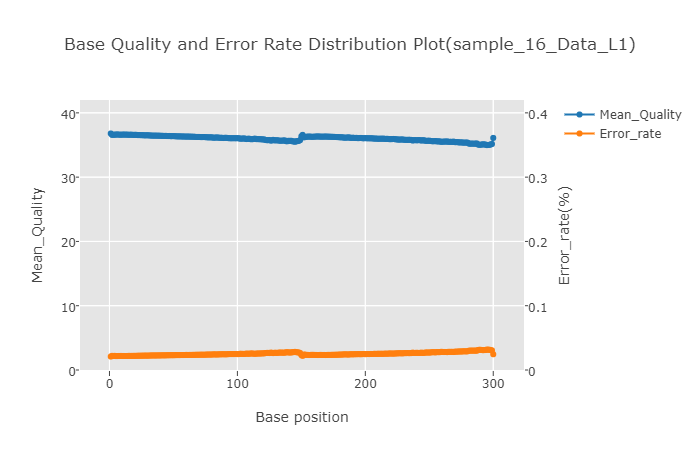


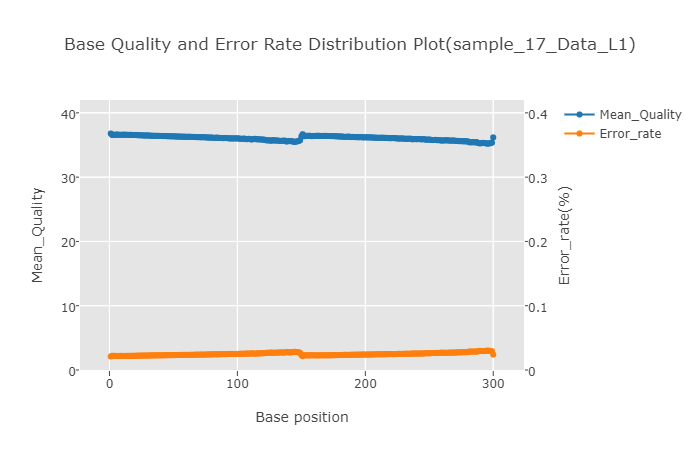

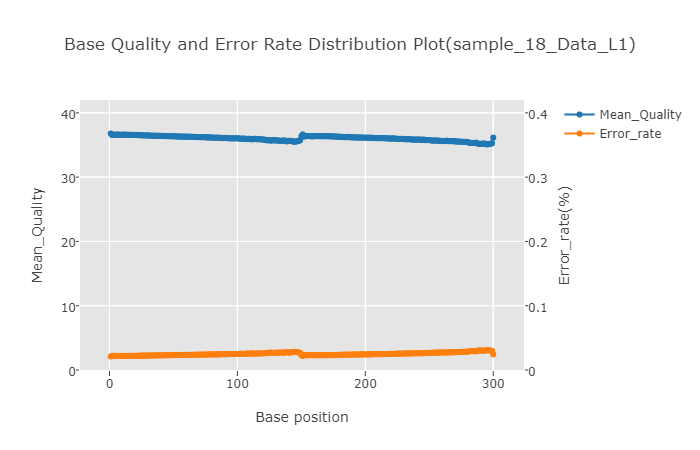


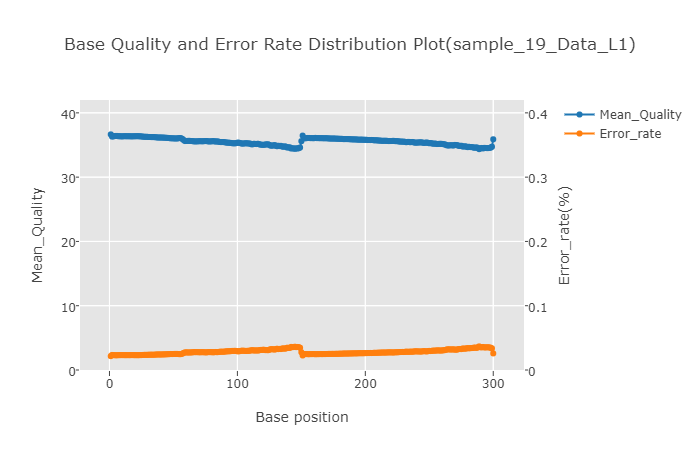

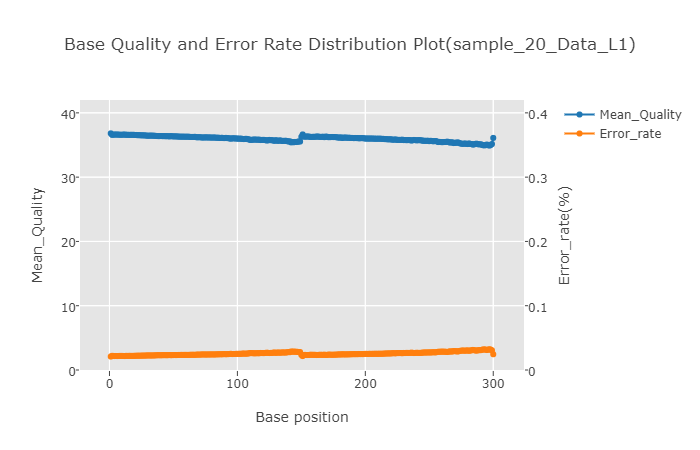


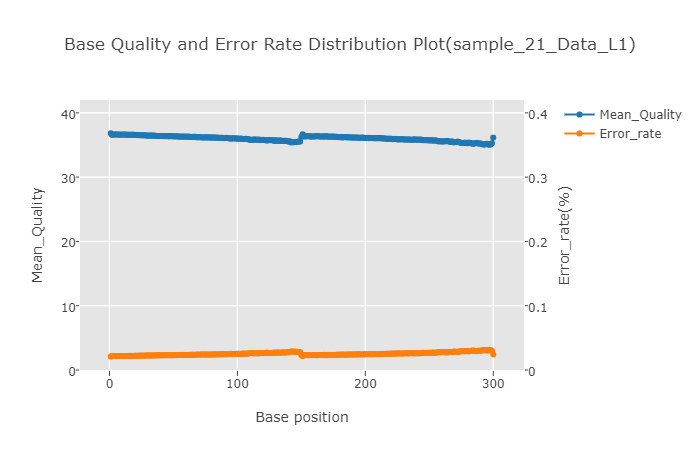

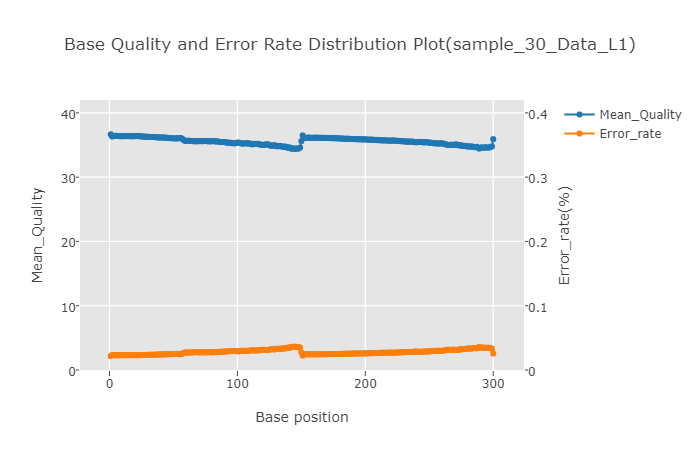


**The distribution of Sequencing Quality and Error Rate**

X-coordinate show base positions. Left half is read 1, right half is read 2.

Left Y-coordinate presents average Q value. Right Y-coordinate presents mean error rate.

**SEQUENCING DEPTH**

**
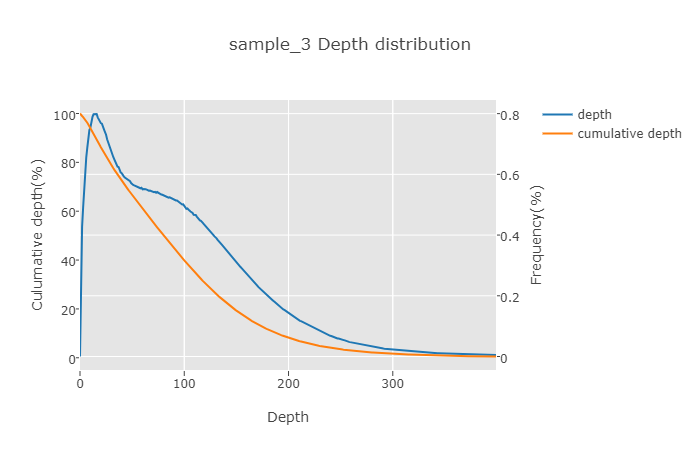
** **
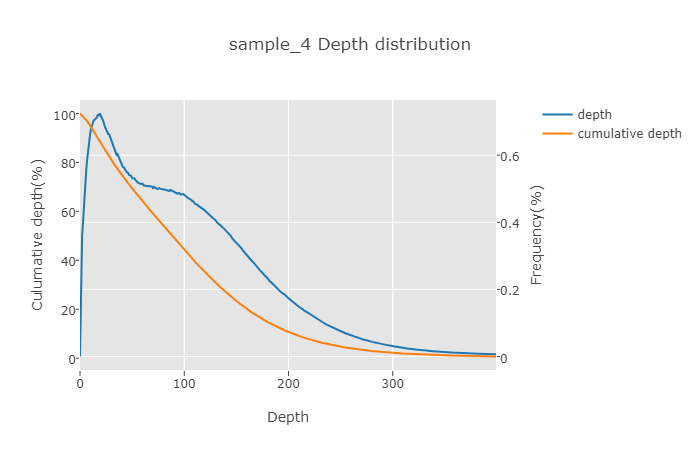
**


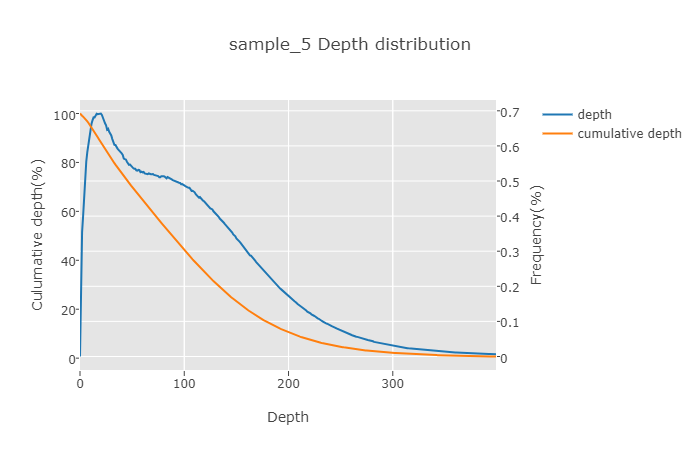

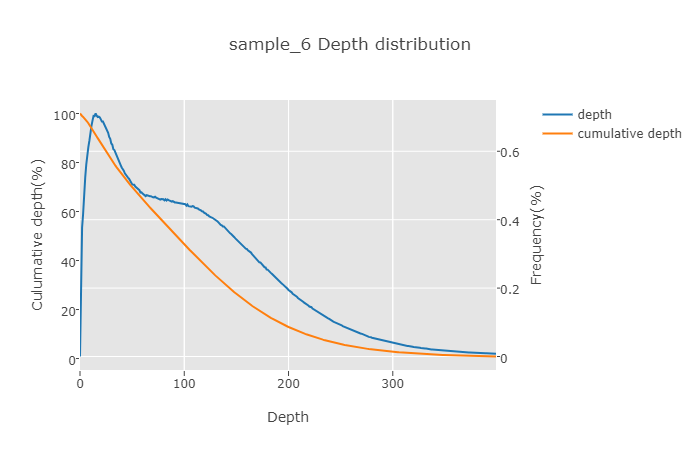


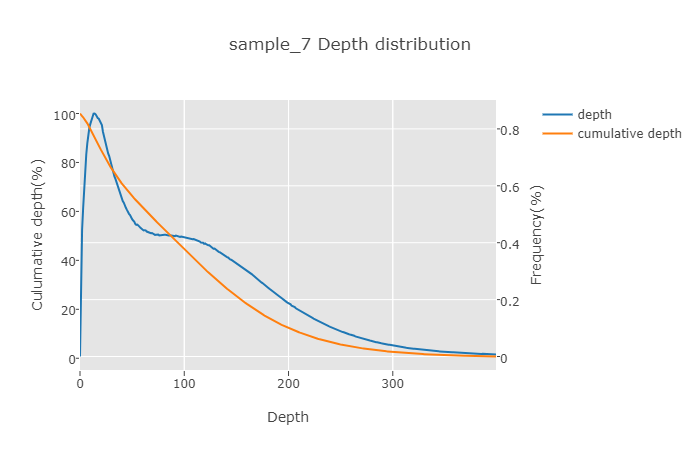

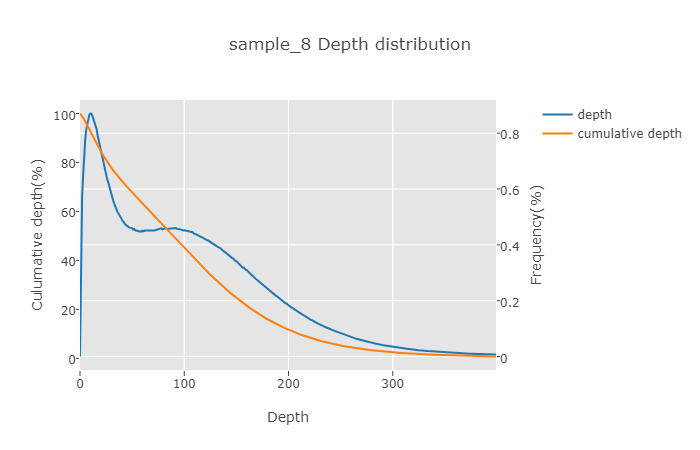


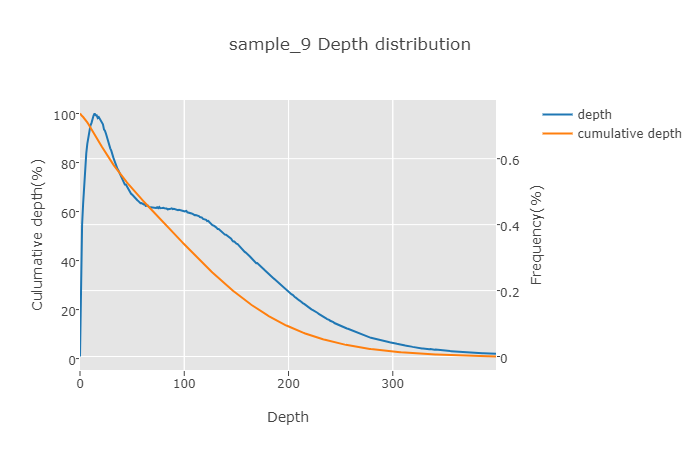

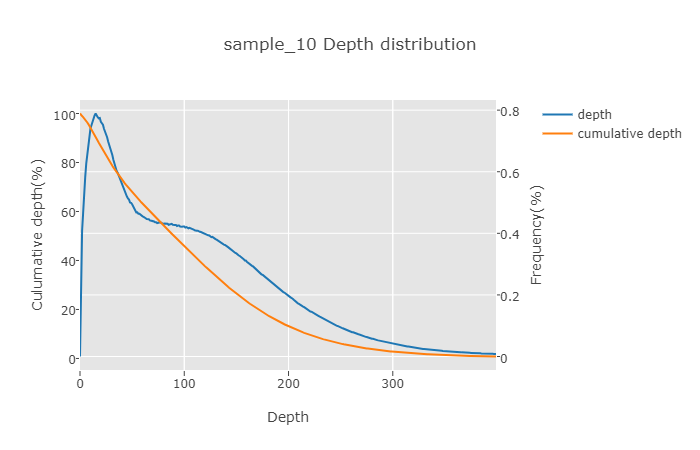


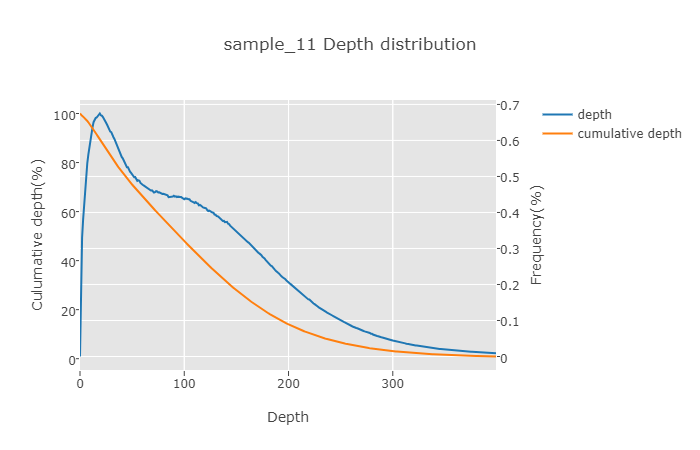

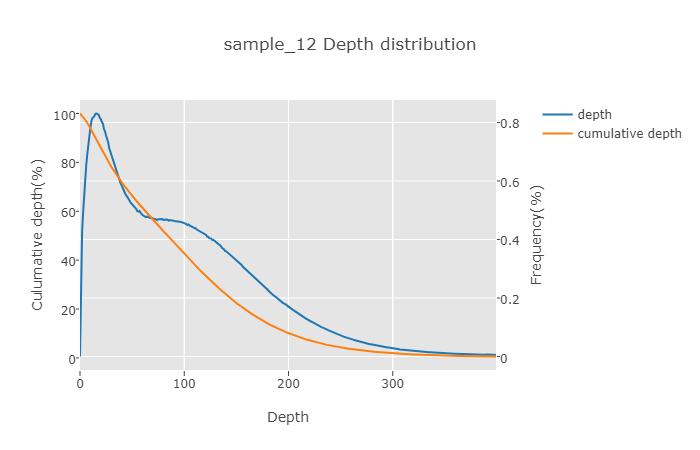


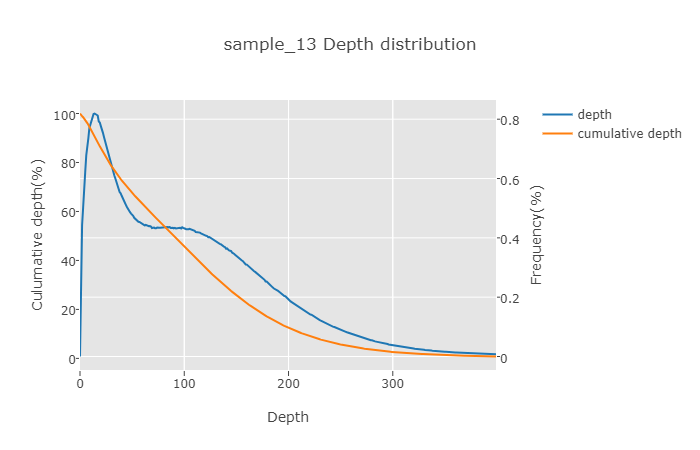

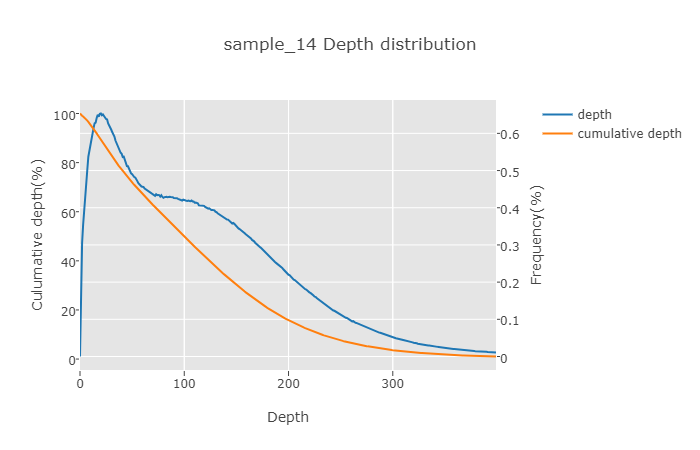


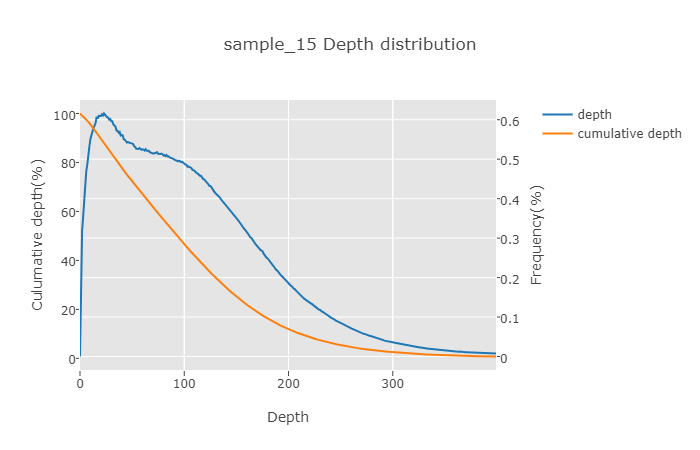

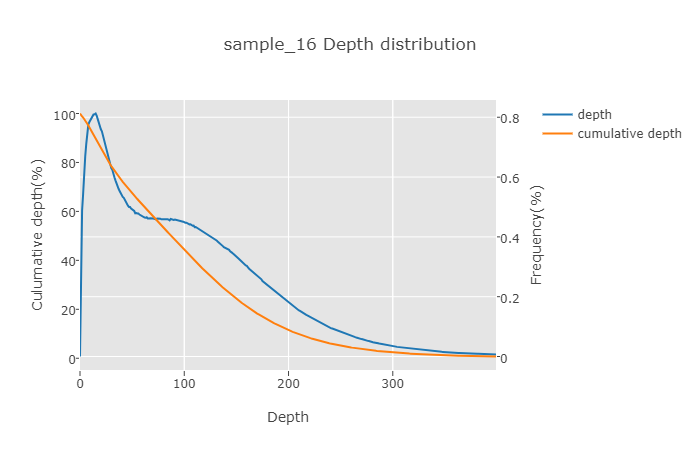


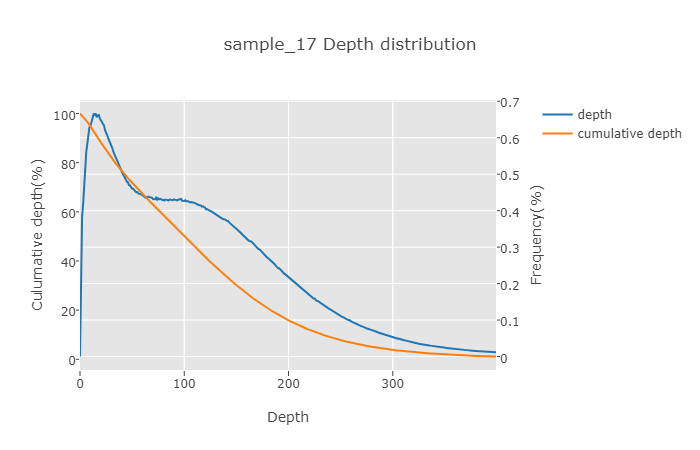

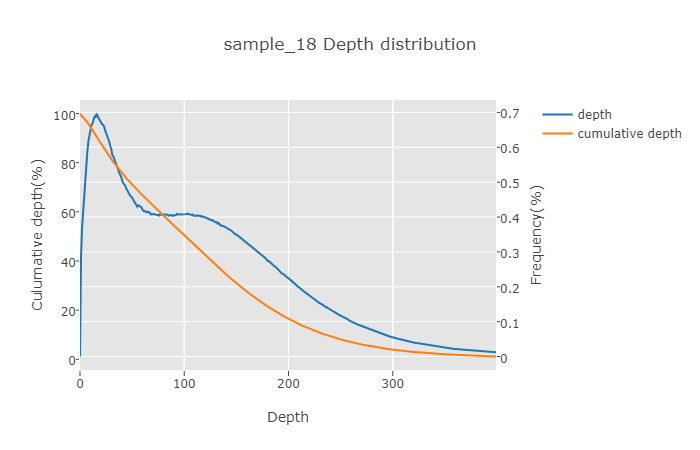


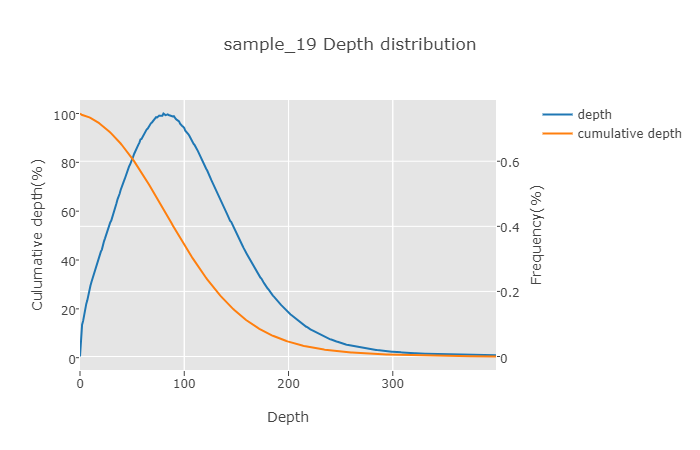

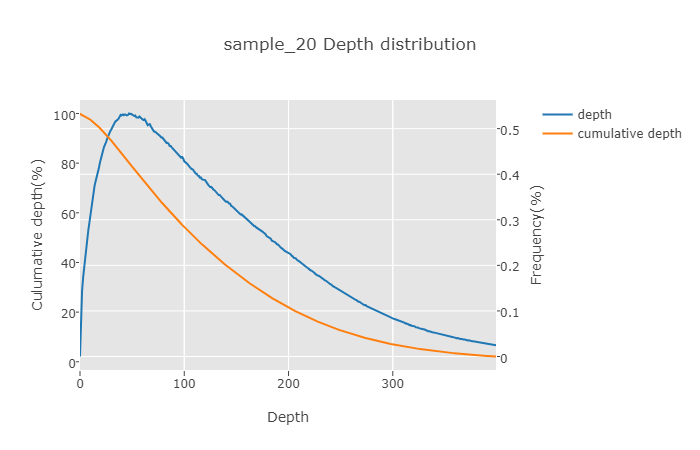


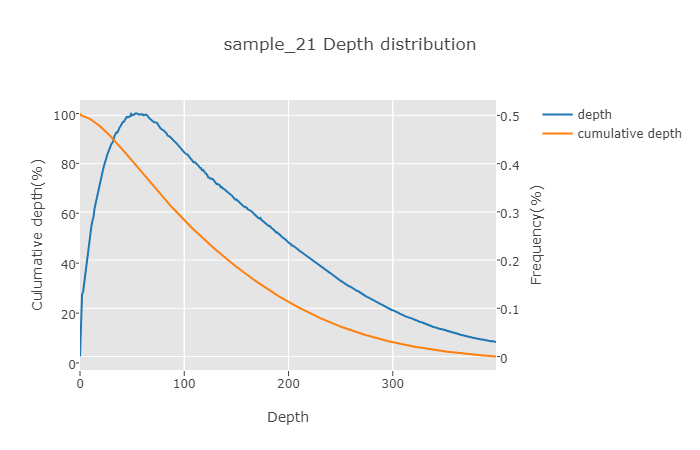

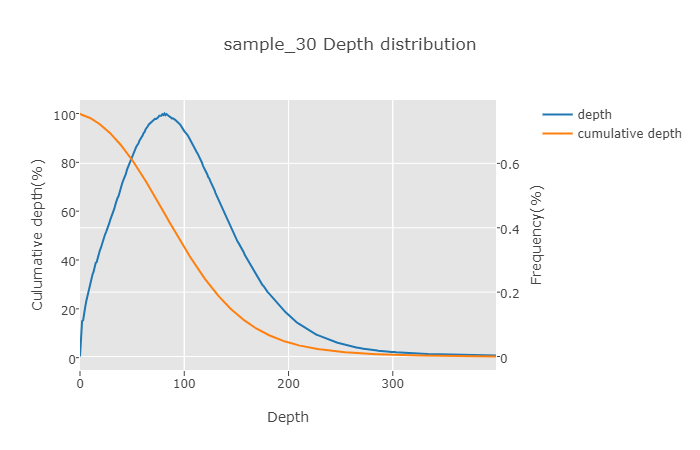


**The distribution of sequencing depth**

X-coordinate presents the sequencing depth of the genome.

Left Y-coordinate indicates the proportion of the corresponding depth in genome. Right Y-coordinate represents the proportion of the corresponding cumulative depth in genome.

**DEPTH AND COVERAGE OF EACH CHROMOSOME**

**
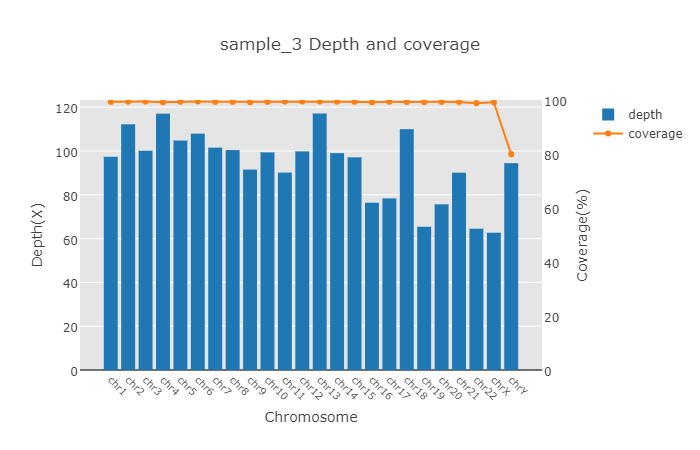

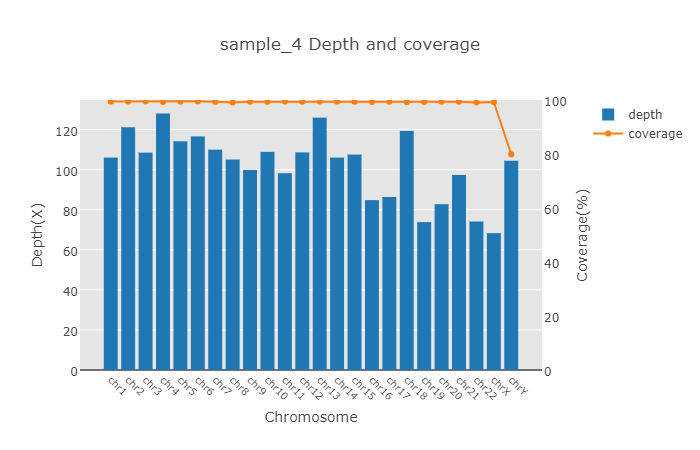
**


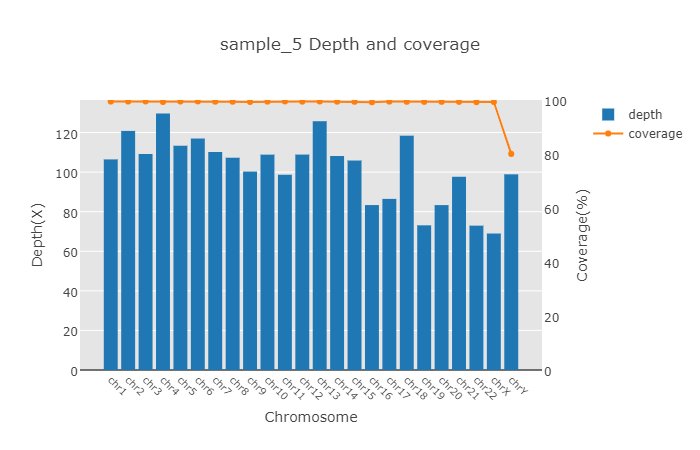

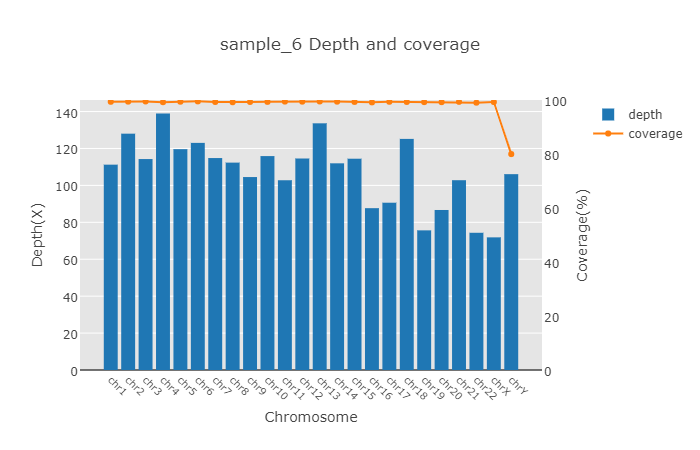


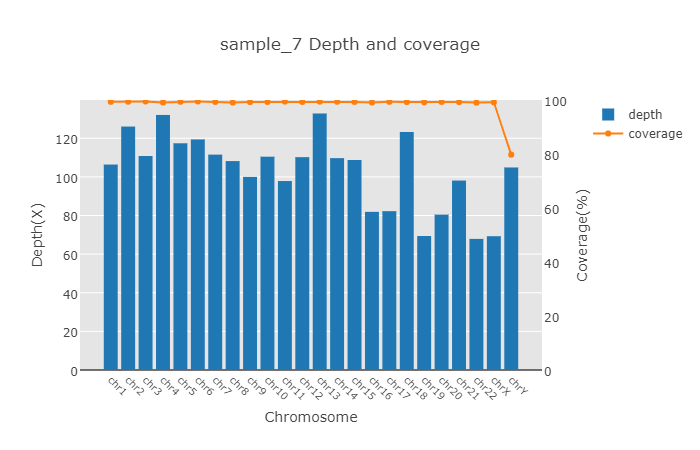

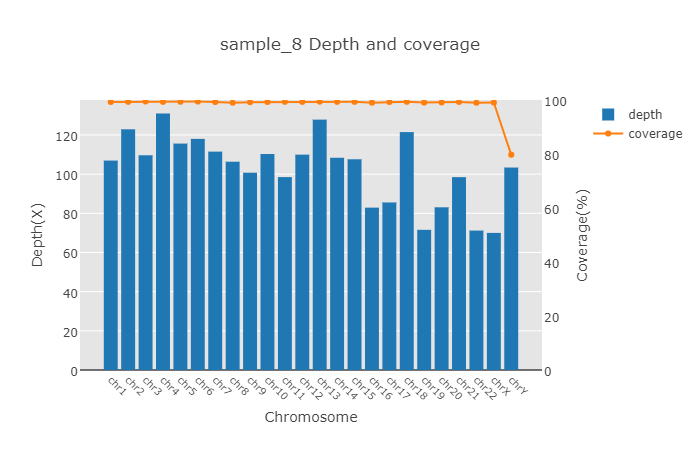


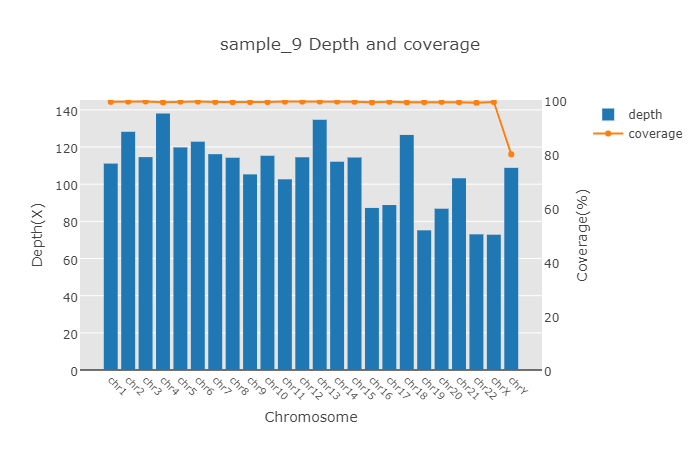

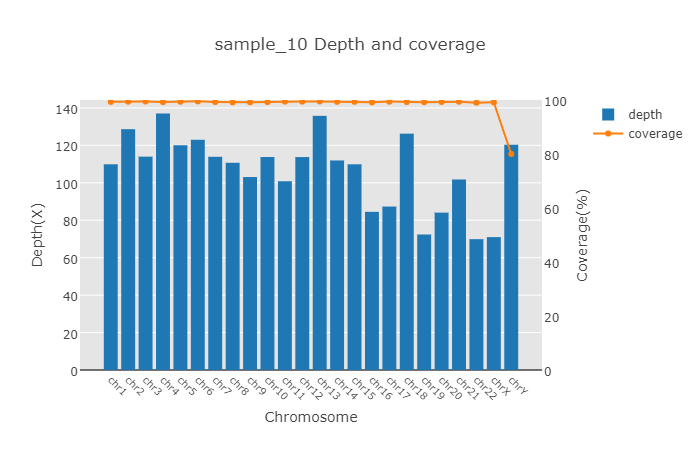


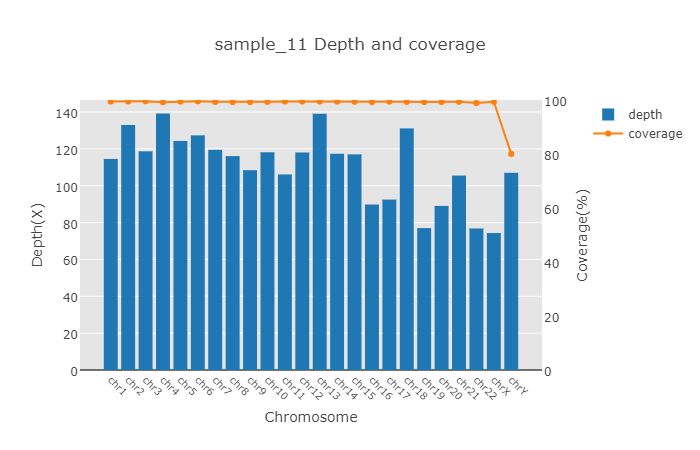

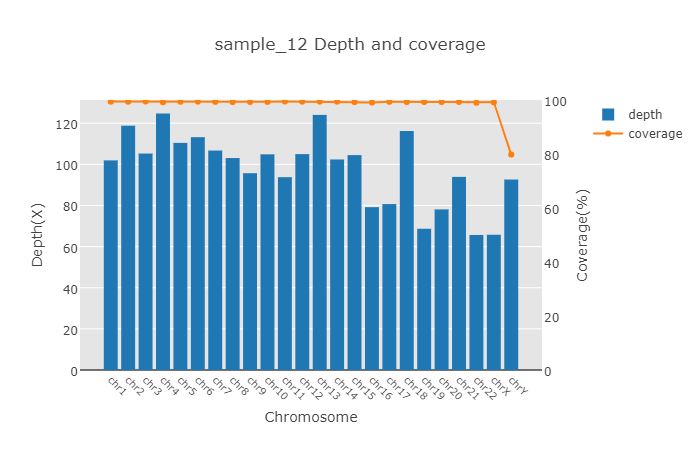


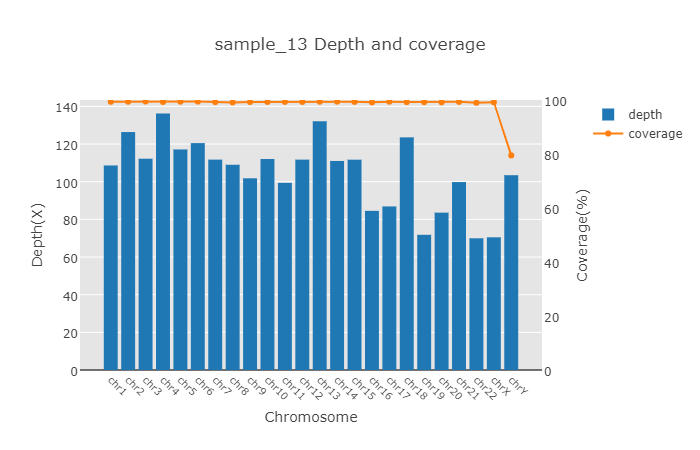

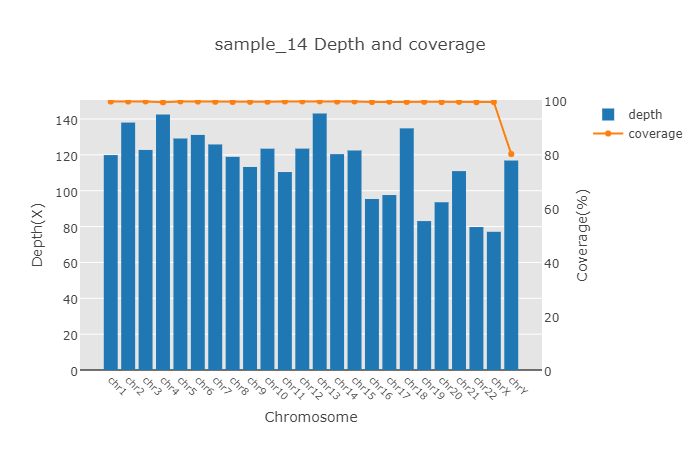


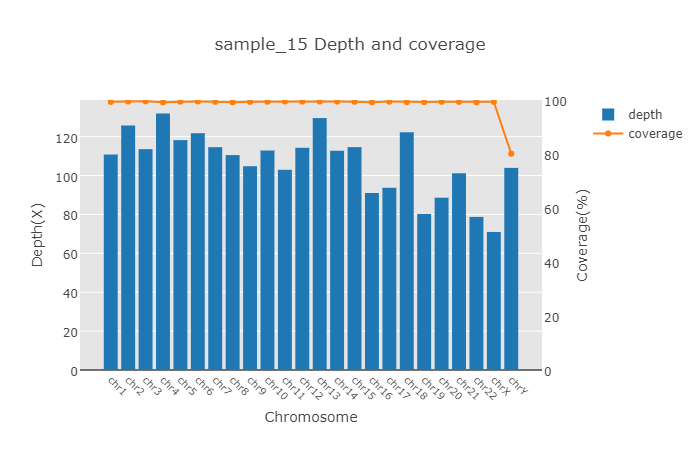

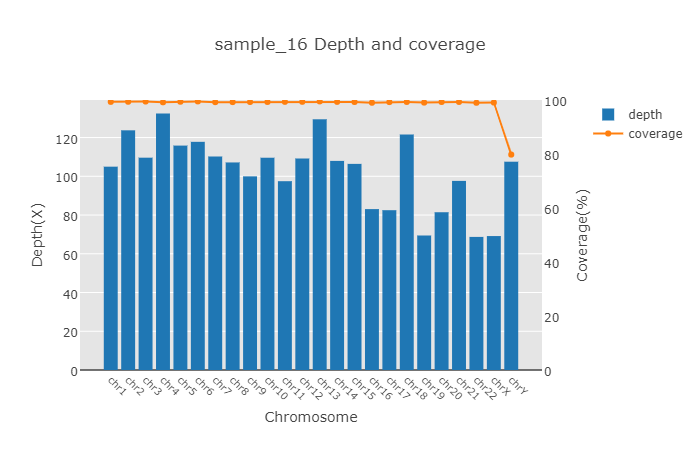


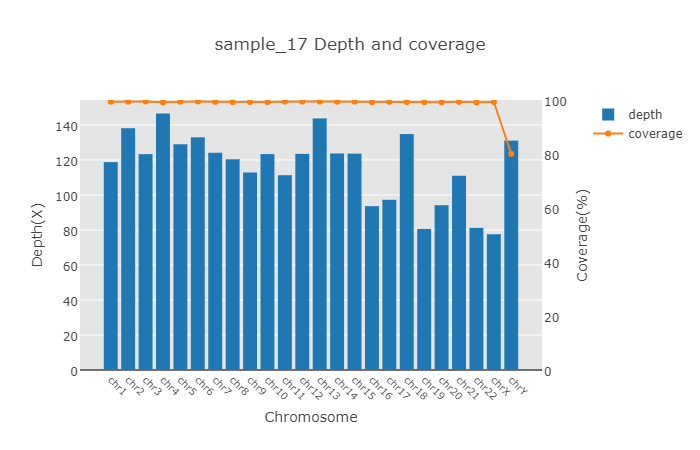

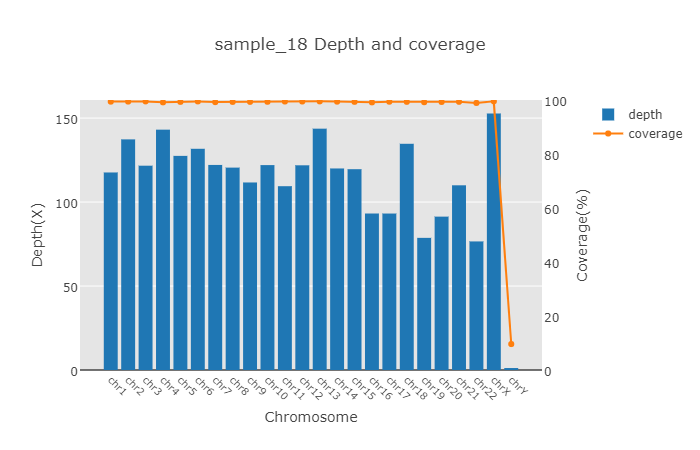


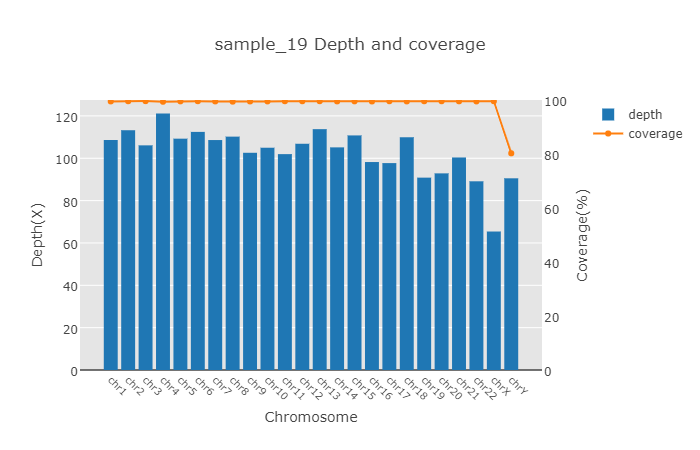

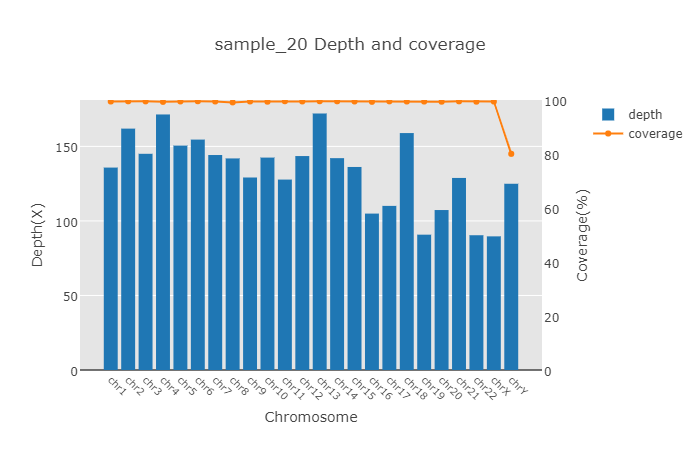


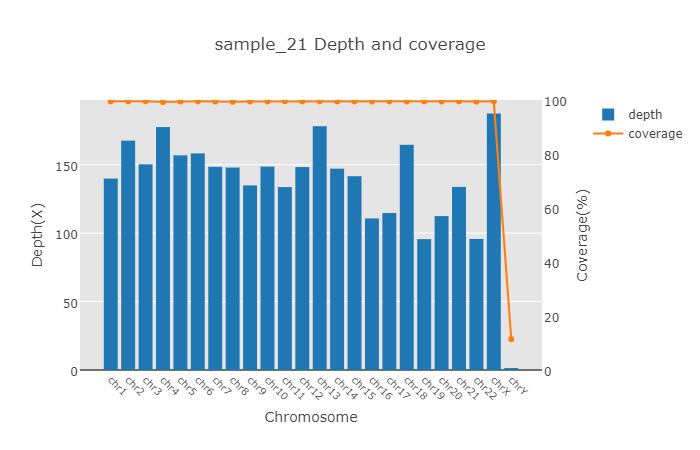

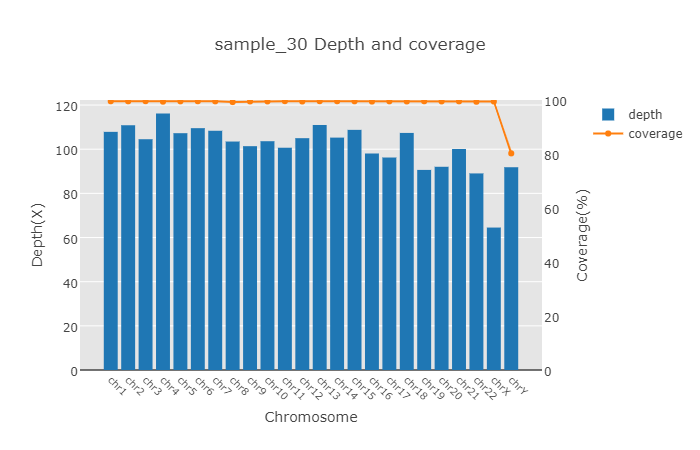


**The distribution of the depth and coverage of each chromosome**

X-coordinate shows chromosome number. Left Y-coordinate shows average sequence depth. Right Y-coordinate shows average coverage of each chromosome.
