# Supplementary material for: Genetic modifiers of response to thalidomide in transfusion-dependent beta-thalassemia patients: a whole-exome sequence analysis
Source: PeerJ. 2025 Oct 7;13:e20038. doi: 10.7717/peerj.20038 (PMC12513377; doi:10.7717/peerj.20038)
Supplement: Supplemental Information 3 [file peerj-13-20038-s003.docx]

**Supplementary file 3:** Search strategy for genes in annotated file.

The genes mutations differentially between excellent responders and non-responders were searched manually in annotated files. As the exome data was very extensive, so a stepwise strategy was devised to avoid missing of any gene and gather as much information form the data as possible. The stepwise scrutiny of exome data in responders versus non responders took about 16 weeks and is as follows:

INDIVIDUAL/SELECTIVE GENES SEARCH

Step 1: Primary, secondary and tertiary modifier genes in thalassemia were searched. Each gene was searched separately and consideration was given to each of the “benign”, “pathogenic”, “uncertain” and “unknown” mutations (list of genes attached at end of file).

Step 2: Genes related to alpha, beta, gamma and delta globin genes were searched from STRING database. Genes from first, second and third shell of interaction were selected from STRING database and each gene was searched separately in exome sequence data for any mutation (list of Genes attached at end of file).

Step 3: Genes that regulate gamma globin expression including genes involved in Nucleosome remodeling and deacetylase (NURD) complex were searched for possible mutations (list of genes attached below).

Step 4: Genes that are expressed in hematopoietic stem cells stepwise at different stages of hematopoiesis mentioned in literature were searched individually ( link of research publication and list of genes attached below ).

SEARCH THROUGH FILTERS

Step 5: “pathogenic” and “likely pathogenic” genes were searched by applying “pathogenic” and “likely pathogenic” filters.

Step 6: “uncertain” genes were searched by applying “uncertain” filter

Step 7: “unknown” genes were scanned using “unknown” filter

For each of the above step, all genes were scanned thoroughly to not miss any mutation.

In order to increase the yield of search, for each of the above step, subsequent filters were applied separately via “exonic”, “intergenic”, “long non coding RNA-exonic”, “promoter” and “UTR”.

The above process was repeated three times in order to avoid missing any mutated gene.

Any difference in mutations between excellent responders and non-responders were noted. The detail of genes was searched in Genecard database. The protein function of the gene was searched from Uniprot database. The involvement of genes in different pathways in the body was searched in KEGG pathway database. This was followed by a thorough literature search using PubMed and Google Scholar search engines to report significance of the mutations and SNPs discovered in our study. In light of mutations in our study subjects and information available about those genes and SNPs, effort was done to hypothesize a model that can explain the enhanced gamma chain synthesis by thalidomide in thalassemia patients.

| **Step-1**  **Genetic Modifier genes that can effect Hemoglobin levels** | **Step-2**  **Gamma and alpha globin related genes (from STRING database)** | **Step-3**  **Genes that effect expression of Gamma gene** | **Step-4**  **Genes expressed in HSC at different stages of hematopoiesis** |
| --- | --- | --- | --- |
| **Primary modifiers:**  HBA1, HBA2, HBG1, HBG2  **Secondary modifiers:**  KLF1, Xmnl1, BCL11A, HBS1L-MYB intergenic area, AHSP  **Tertiary modifiers:**  HFE, GTSM1, APOE, HLA, COLIA-A2, TGFB1, UGT1A1 | **Gamma globin related:**  NFE2, GATA1, TAL1, HBD, MAFG, MAFF, MAFK, HBE1, ZFPM1, ATF4, HP, RPS7, RPS12, IKZF1, HDAC1, RPS27A, HPX, MYB, EP300, FOXP3, HRI, DNMT1.  **Alpha globin related genes:**  UBP1, TFCP2, PHC2, PRMT6, MTHFD2, ANKRD29, ARHGAP21, EEF2, RPS15A, RPS9, RPL18A, RPL35, RPL38, | **NURD complex related:**  HDAC1, HDAC2, CHD4, CHD3, MTA2, GATAD2A, GATAD2B, RBB4, RBBP7, RCOR1, SNAI1, MBD2, RB1, HAT1, RBC2, SOX6  **Gamma globin silencing genes:**  HBS1L, MYB, TR2(NR2C1), TR4(NR2C2), FOG1, PDE7B, FOP, C1ORF77, TGF-B, XKLF,DR-1, TRP, GT6, SOX6,BRG1, FKLF, SIN3A, CP2, SSP, PP2A, CTIP1, MBD,MTA1,MTA2,MBD3, RBP7,RBBP4, AR1D1A, AR1D1B, ARID2, SMARCE1, DPE2, SMARCE2, ARID2, SMARCA2, SMARCA4, LSD1, NFY, LIN28B, SOCS3, KRAS, CBL, NF1, PTPN11 | DOI links of the two manuscripts :  [**https: //doi.org/10.1189/jlb.0107014**](https://doi.org/10.1189/jlb.0107014)  10.3389/fcell.2022.664261 |
