# Supplementary material for: Genetic modifiers of response to thalidomide in transfusion-dependent beta-thalassemia patients: a whole-exome sequence analysis
Source: PeerJ. 2025 Oct 7;13:e20038. doi: 10.7717/peerj.20038 (PMC12513377; doi:10.7717/peerj.20038)
Supplement: Supplemental Information 4 [file peerj-13-20038-s004.docx]

Supplementary File 5: Discovered mutations in Exome regions as viewed in Integrated genome viewer. Mapping done with GRCh37- hg19

| **Gene** | **Non responders** | **Excellent responders** |
| --- | --- | --- |
| NPNT | 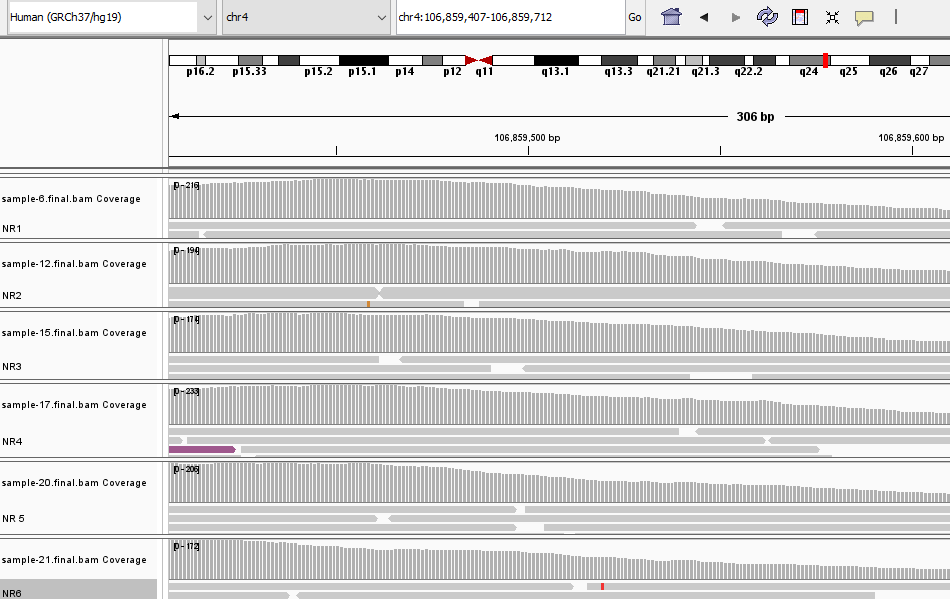 | 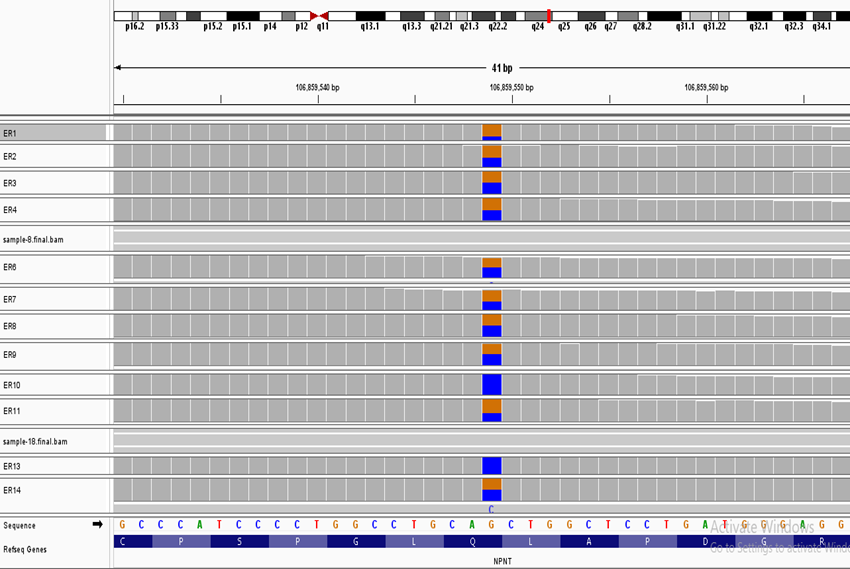 |
| BMP3 | 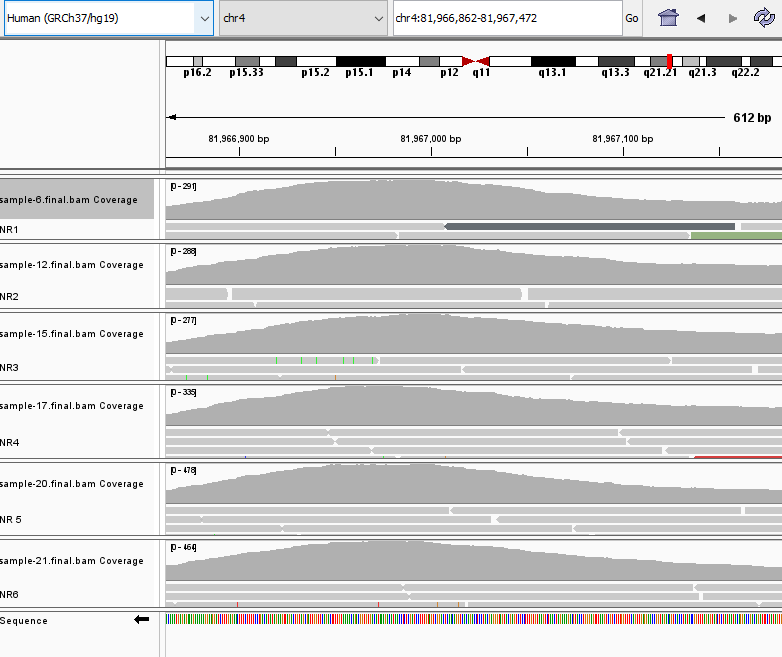 | **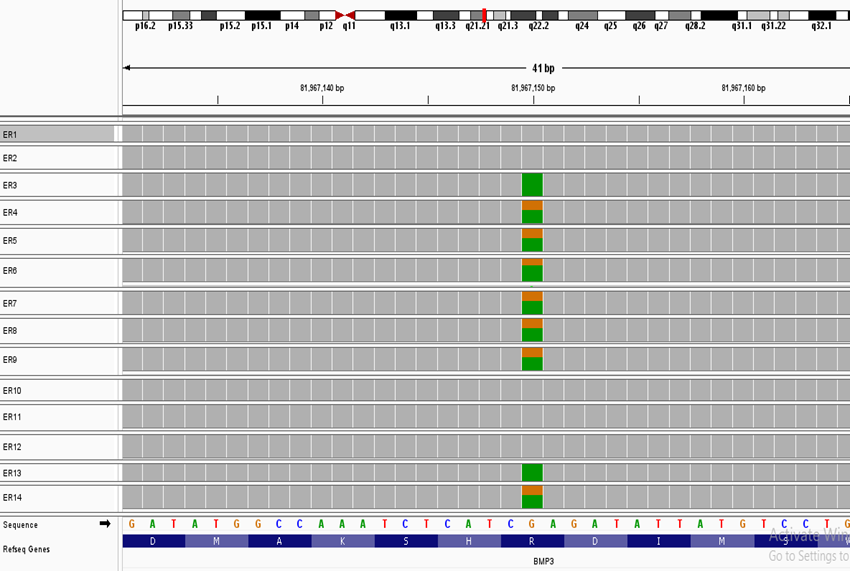** |
| CHD4 | 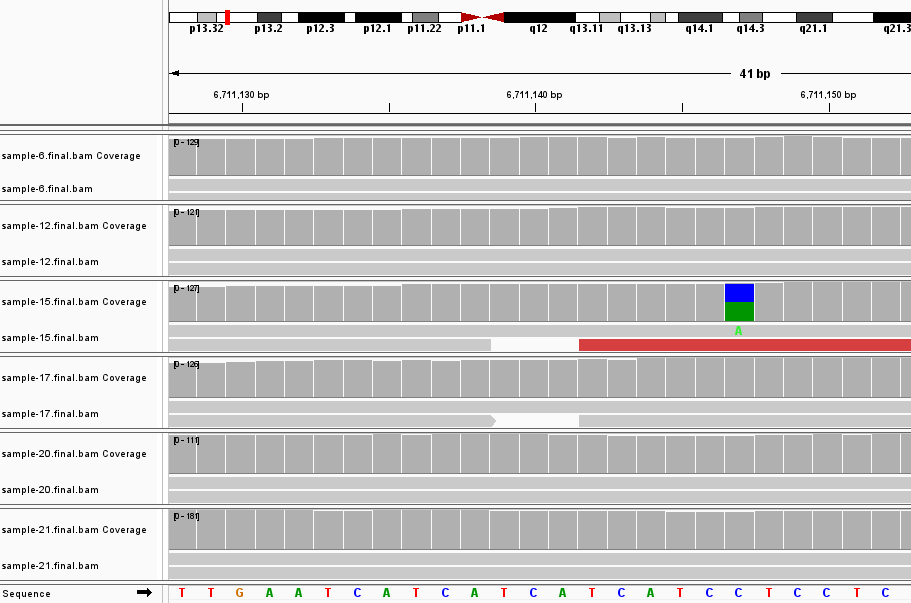 | **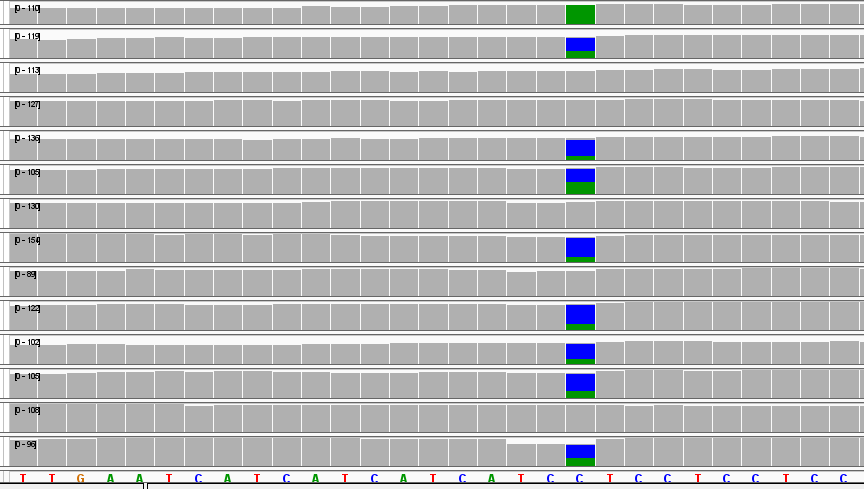** |
| MAPK4 | 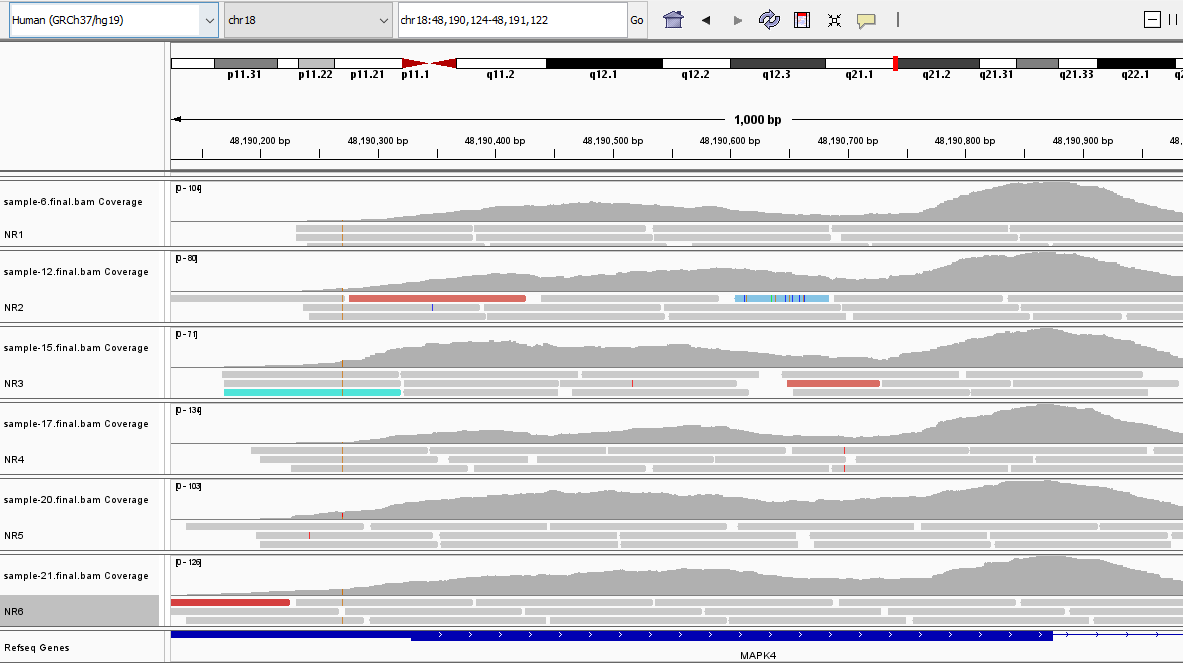 | **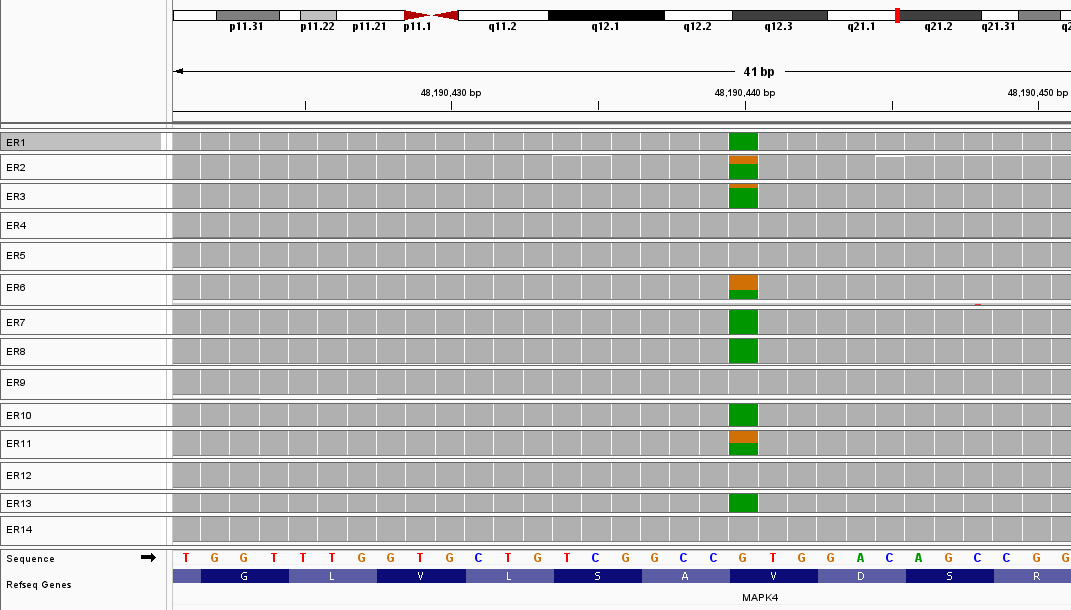** |
| TAF1C | 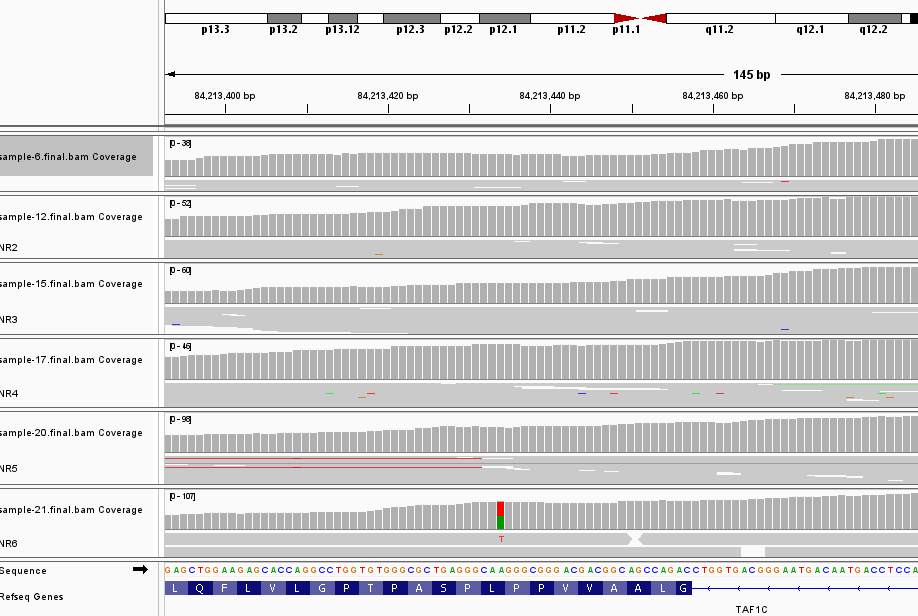 | **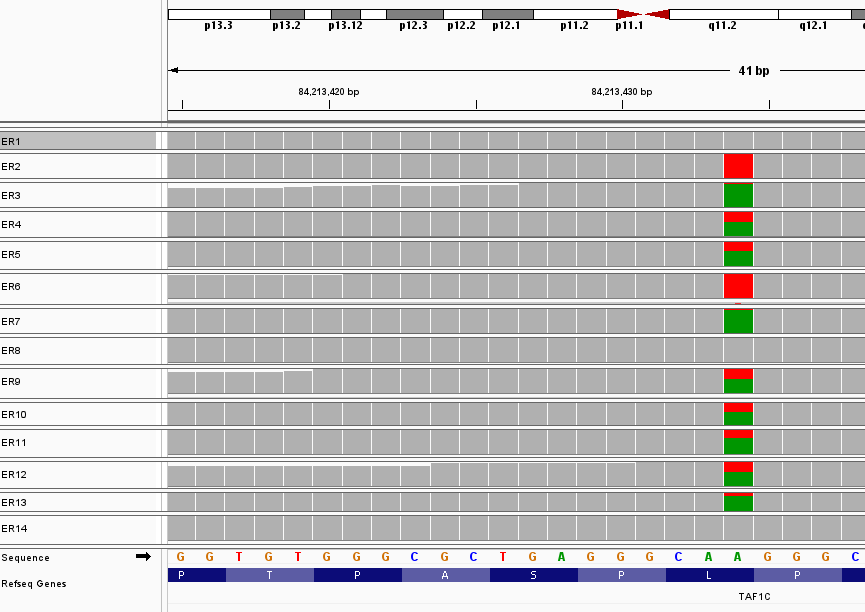** |
| INPP5D | 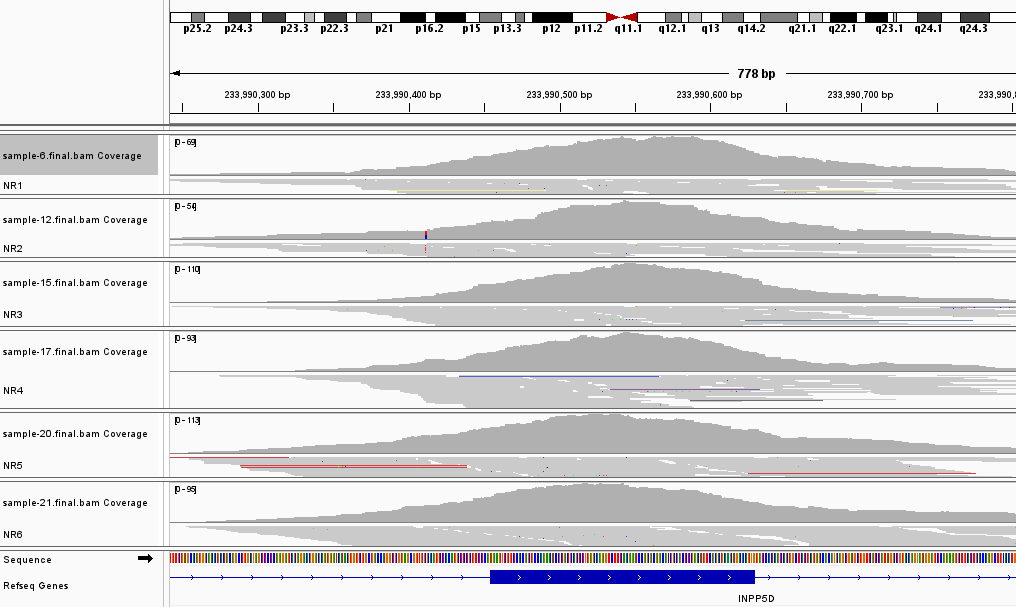 | **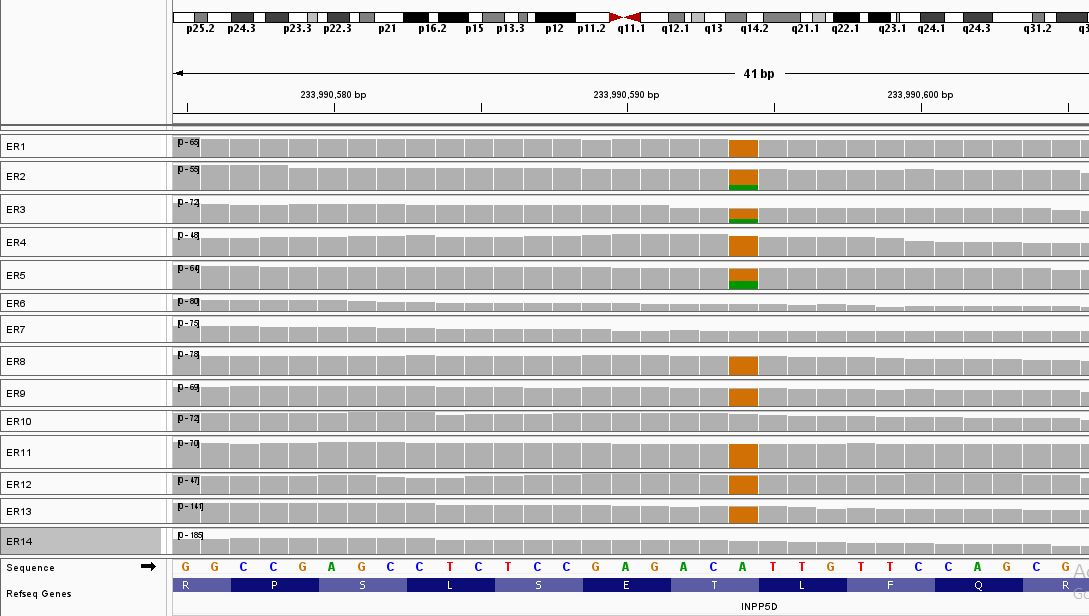** |
| ACP1 | 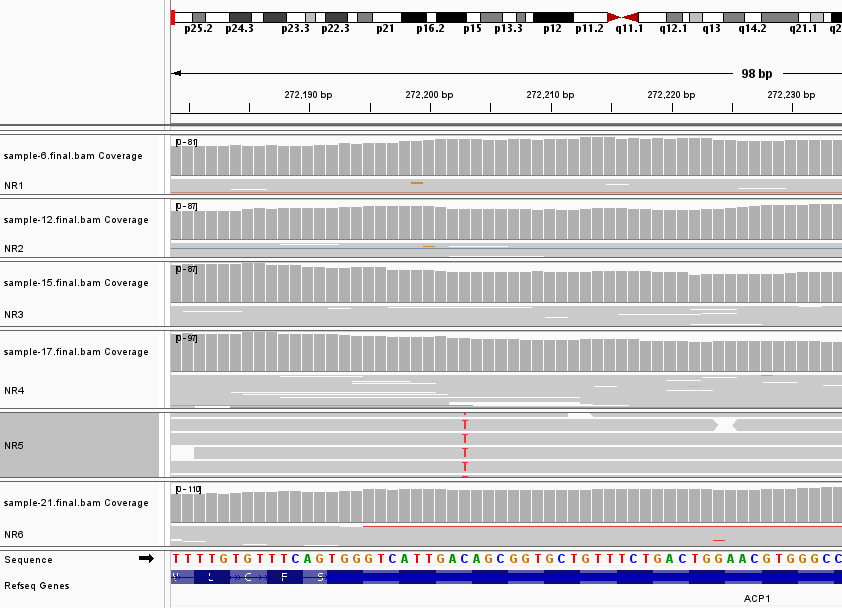 | **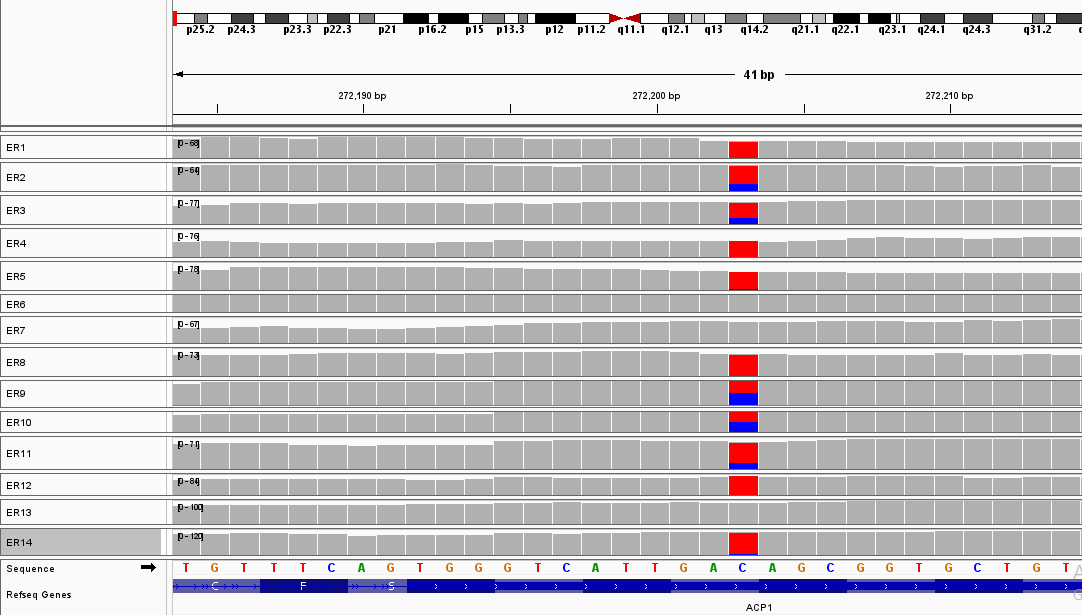** |
| LGR6 | 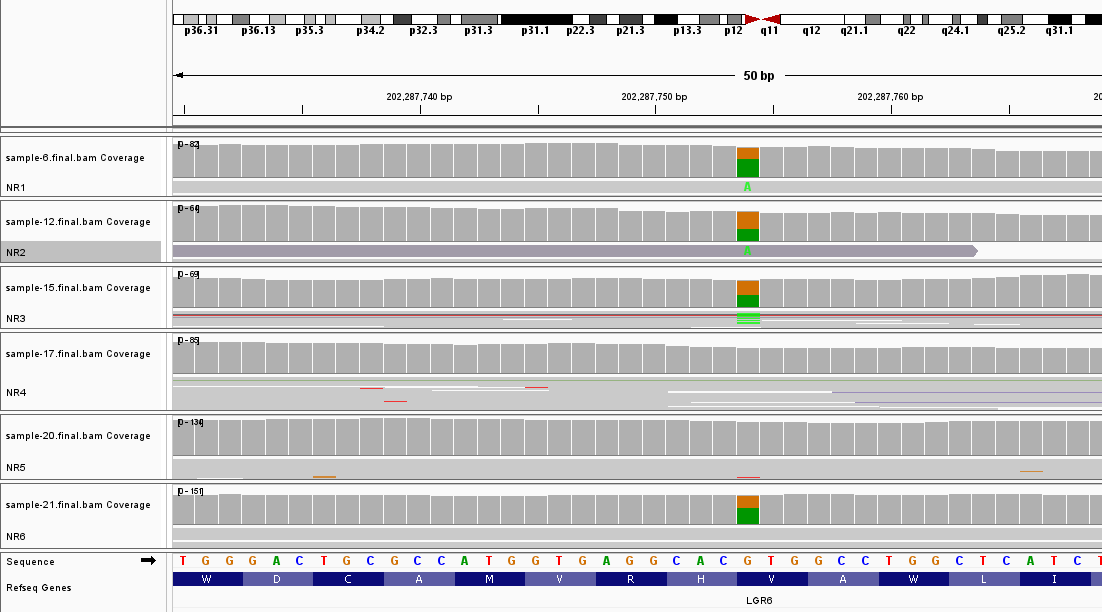 | **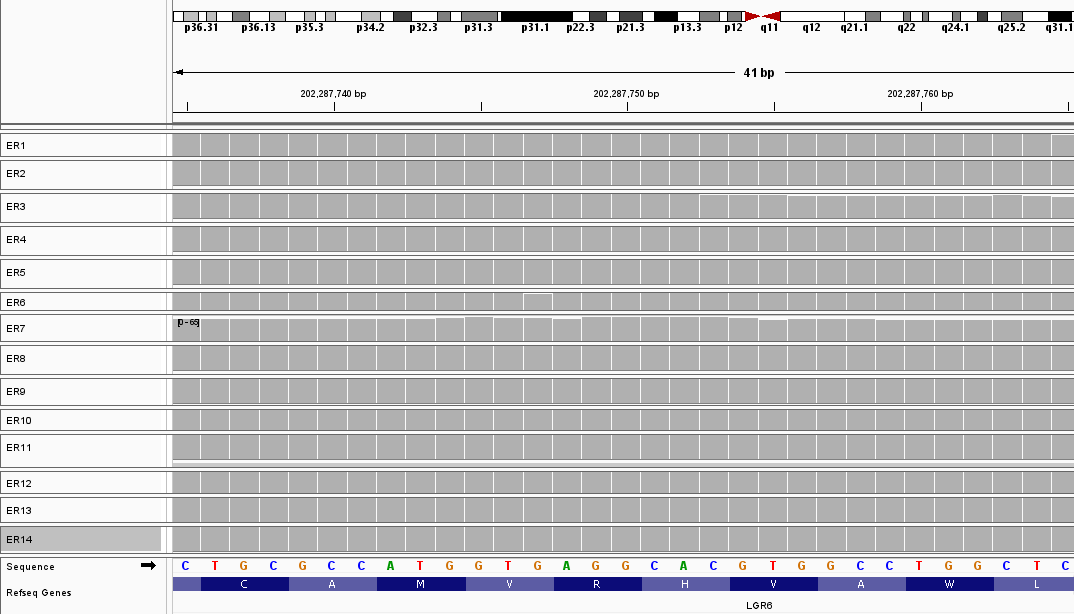** |
| HHAT | 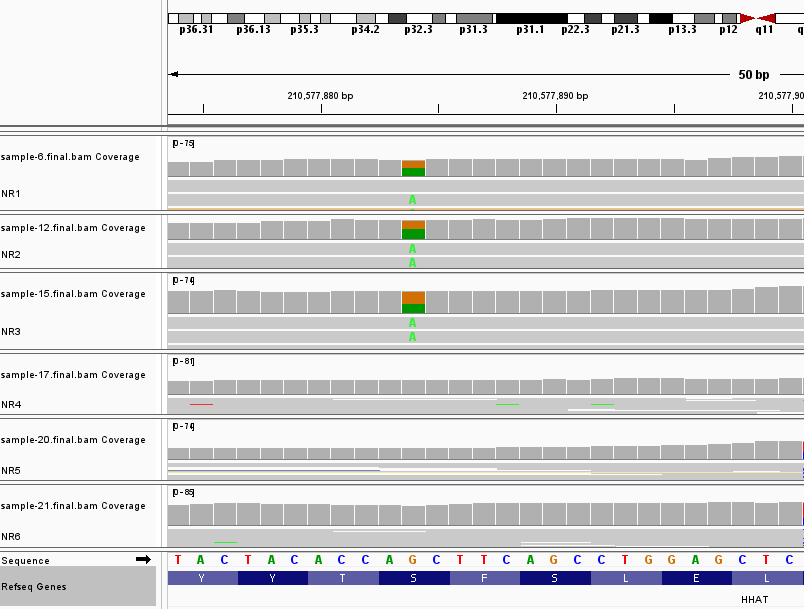 | **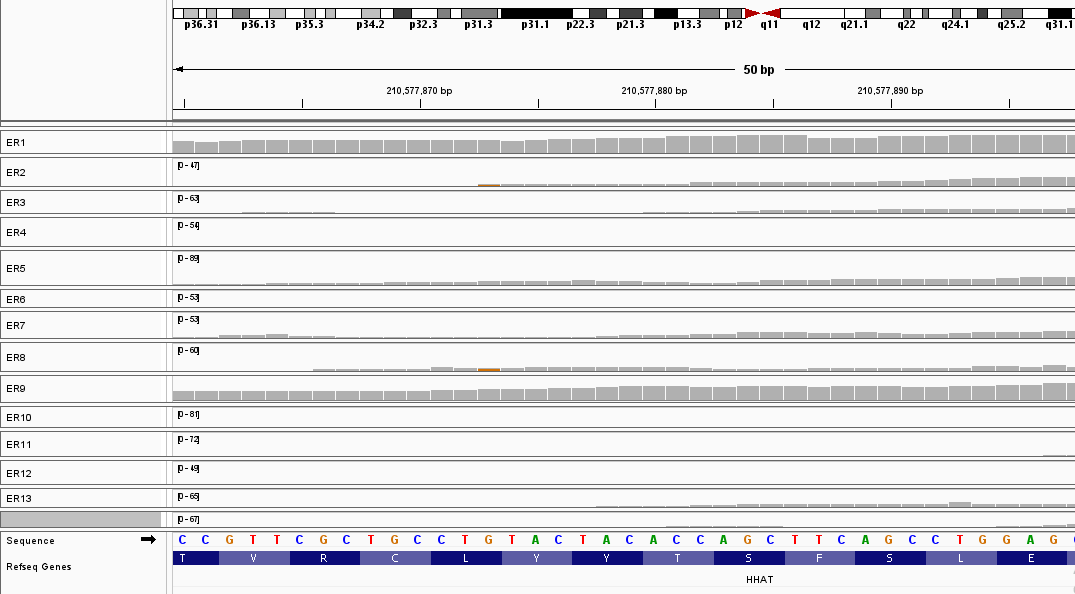** |
| CHI3L1 | 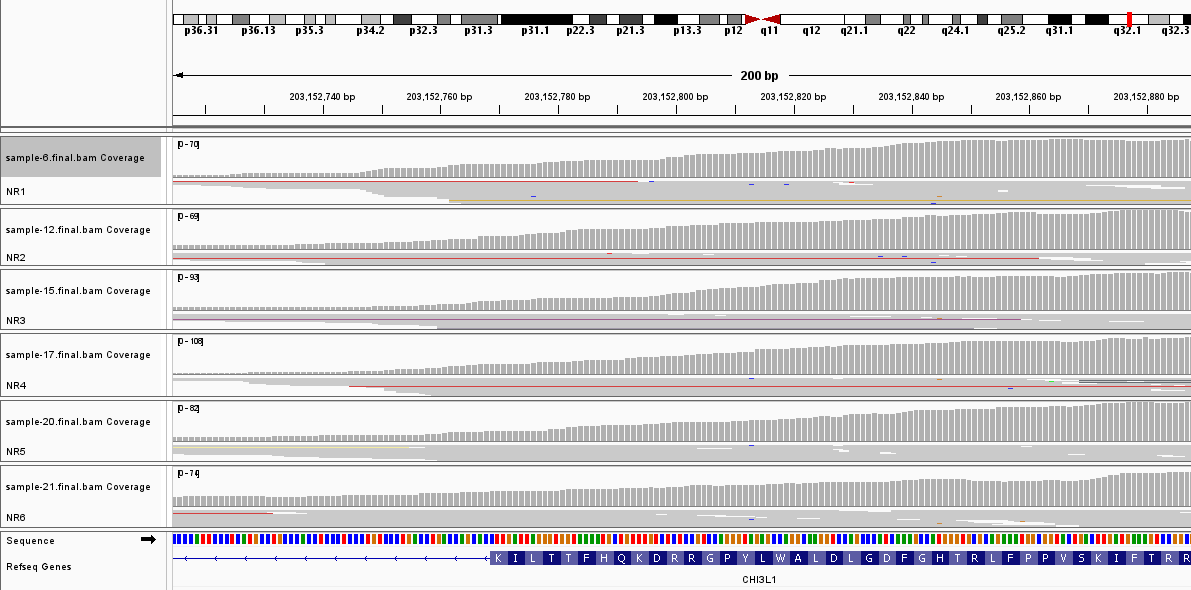 | **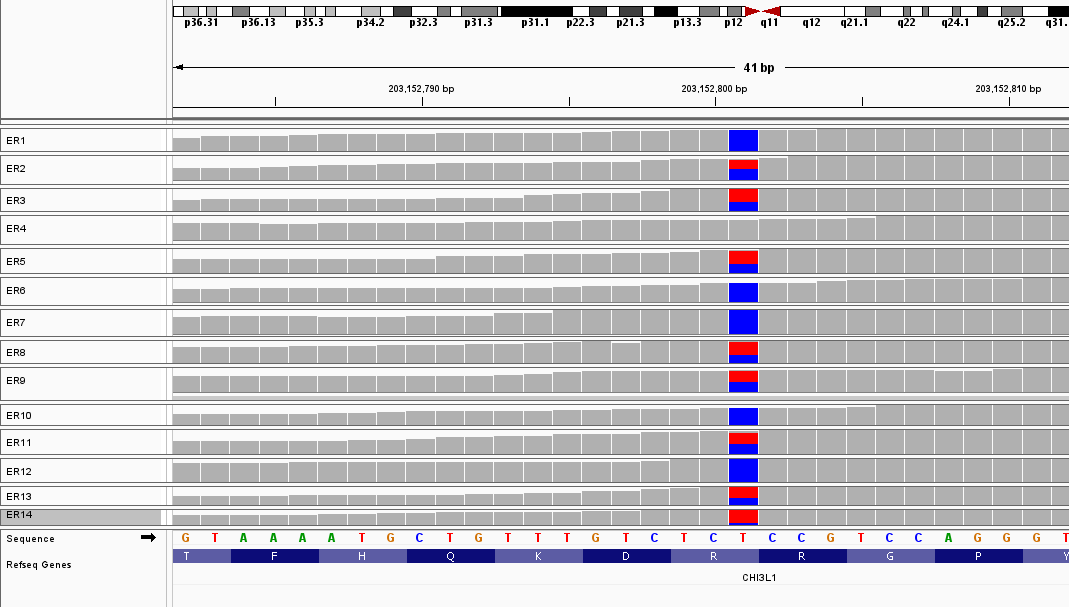** |
| KHNYN | 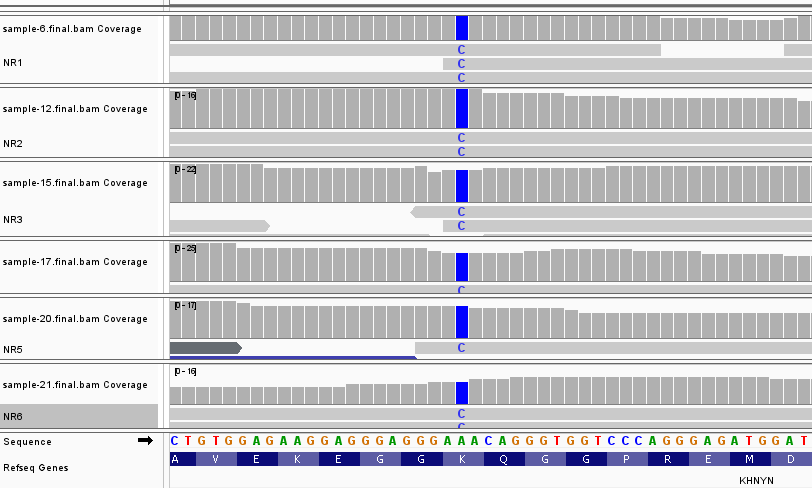 | **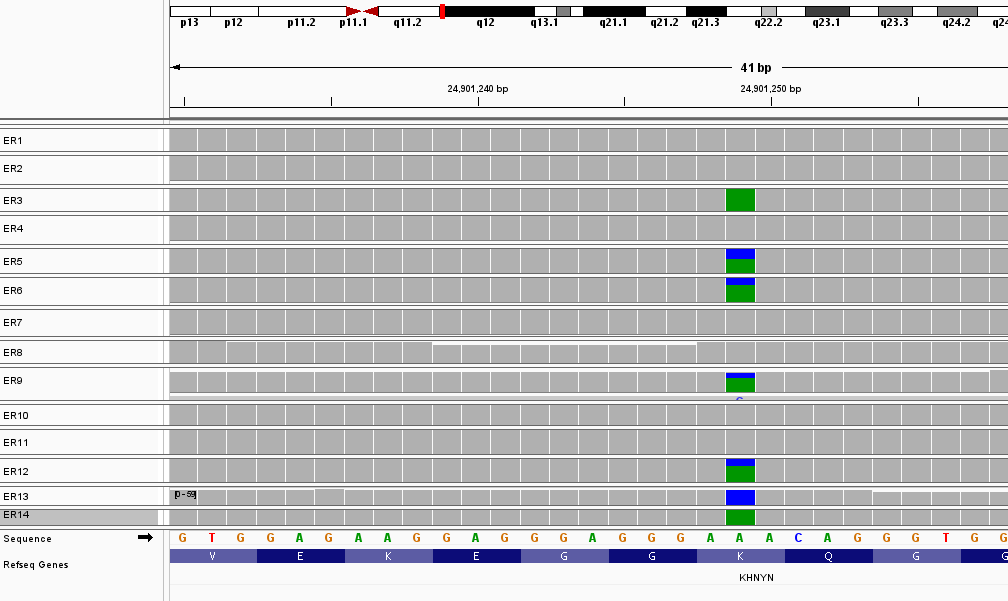** |
| ZNF208 | 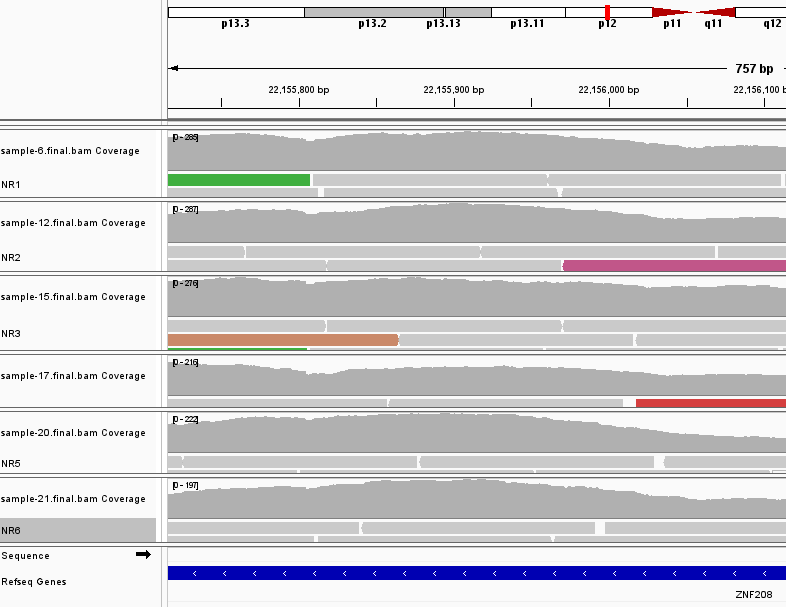 | **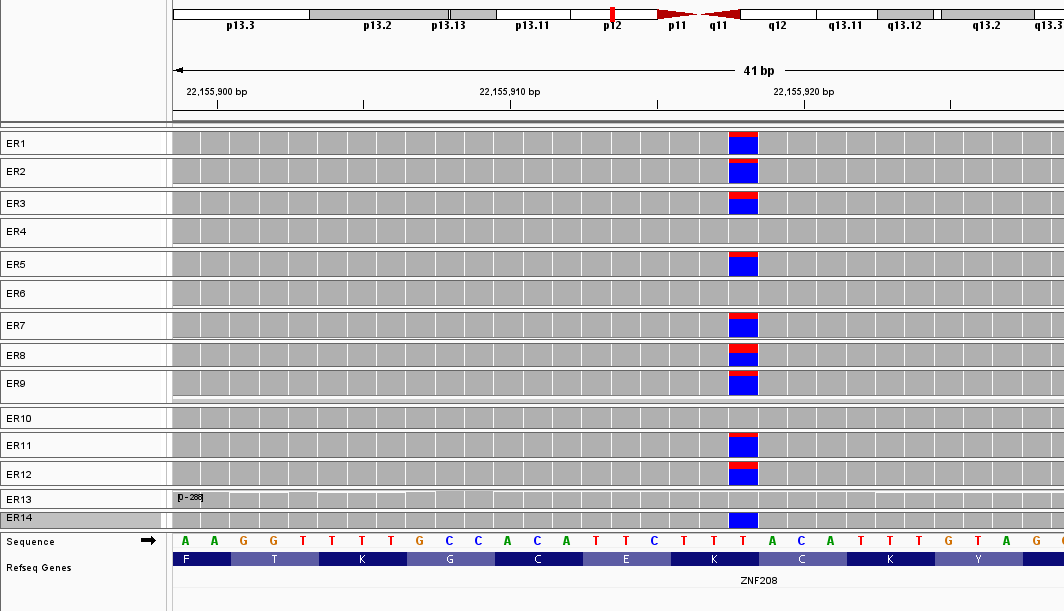** |
| MYH15 | 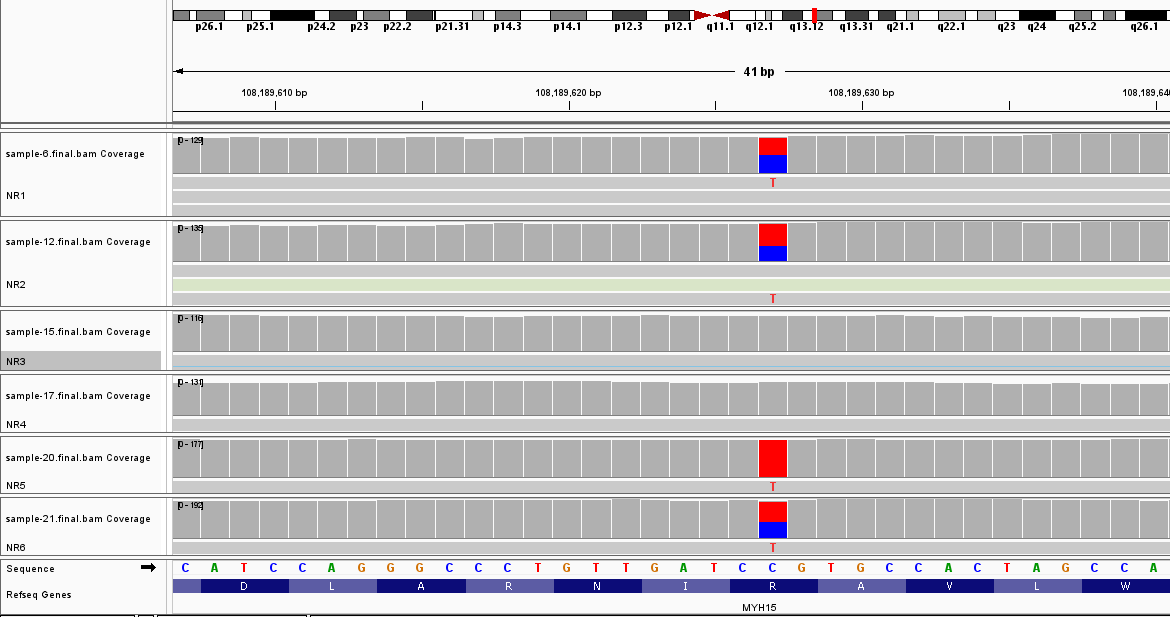 | **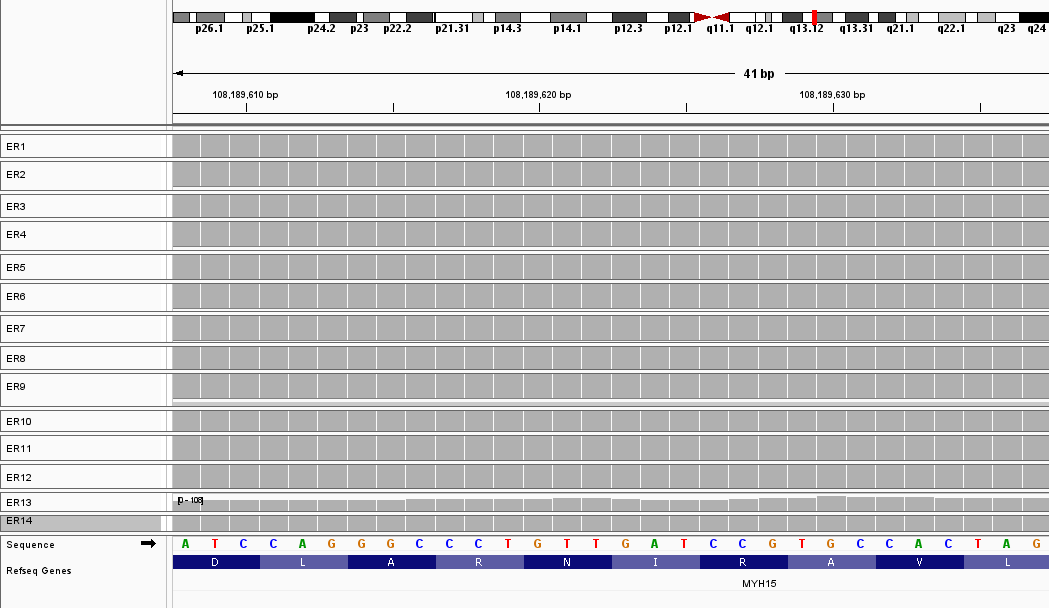** |
| RESF1 | 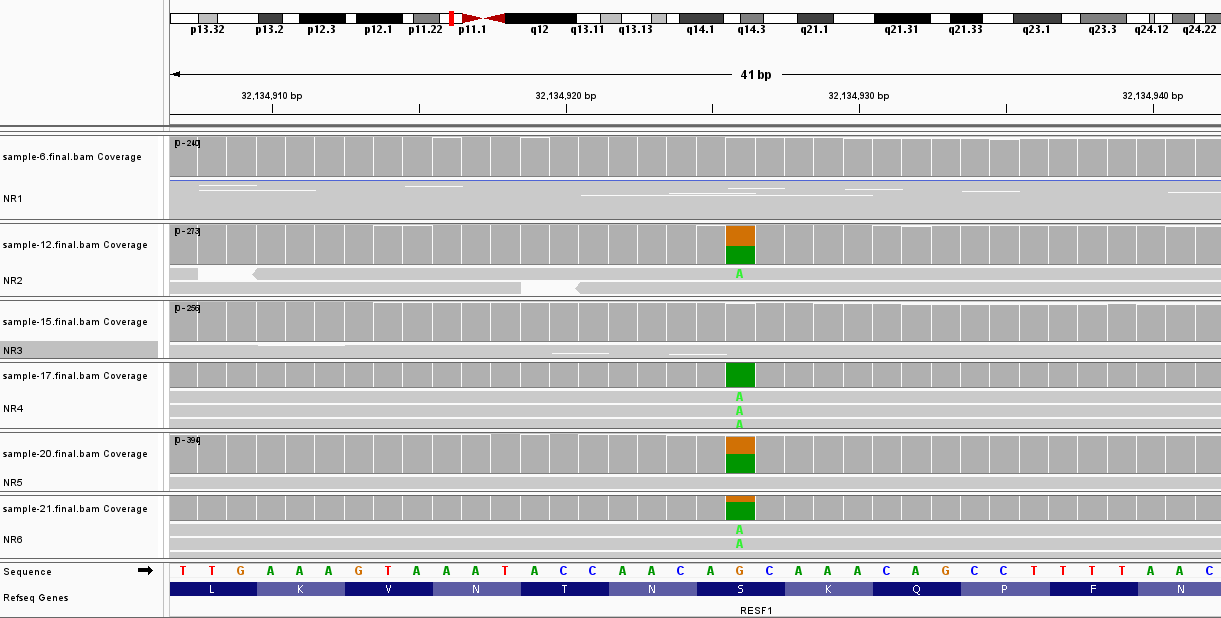 | **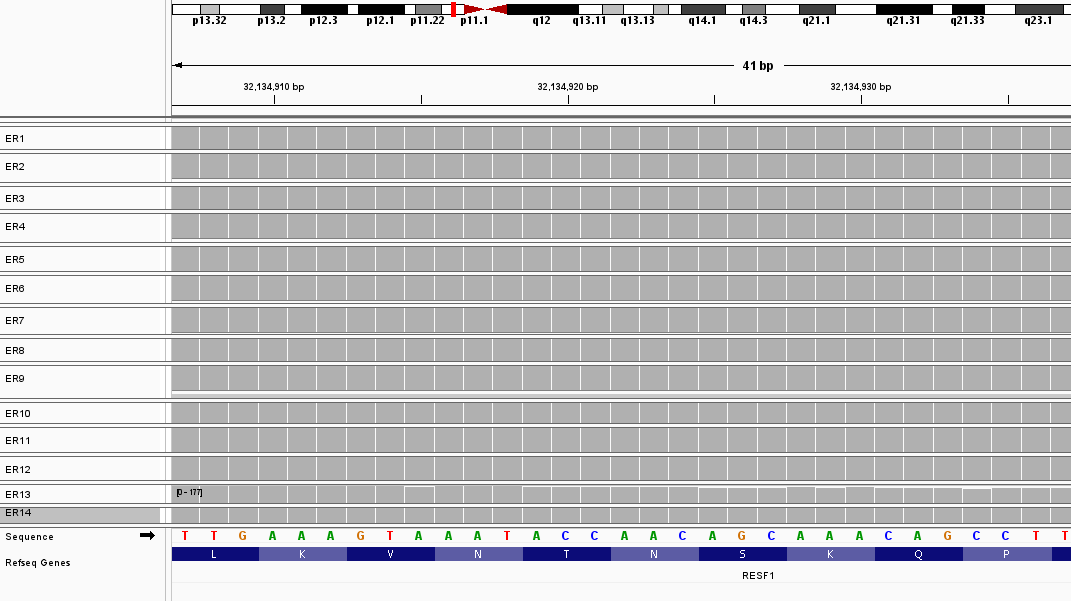** |
| BIRC2 | 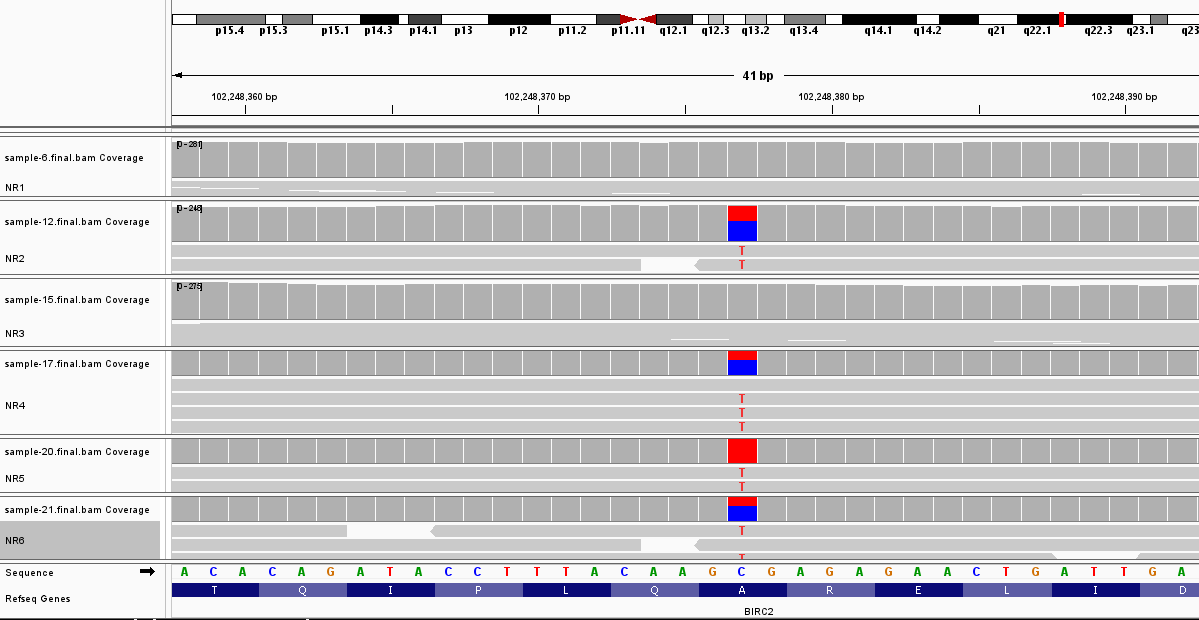 | **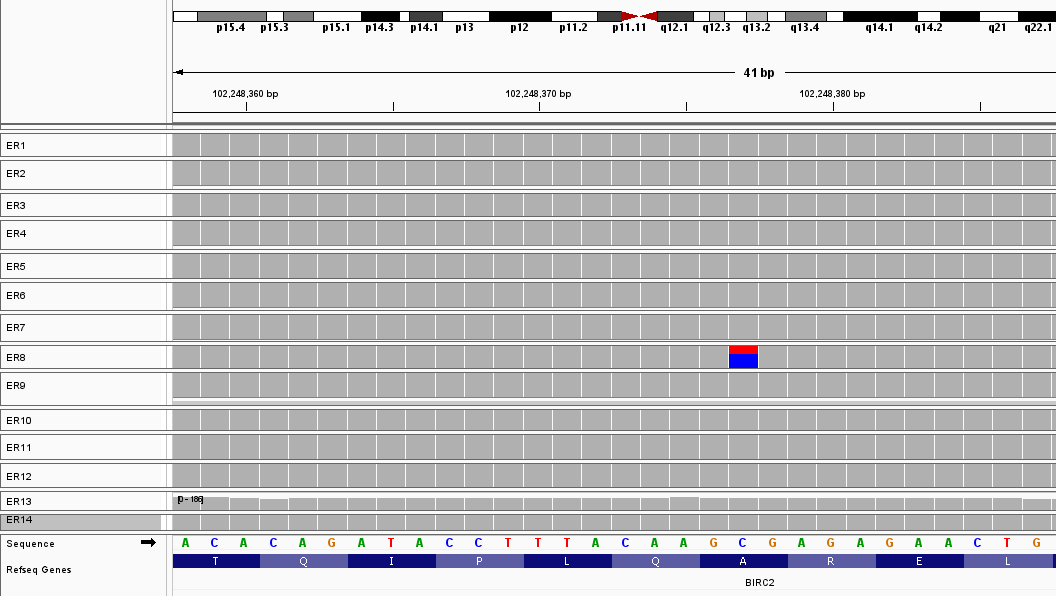** |
| PM20D1 | 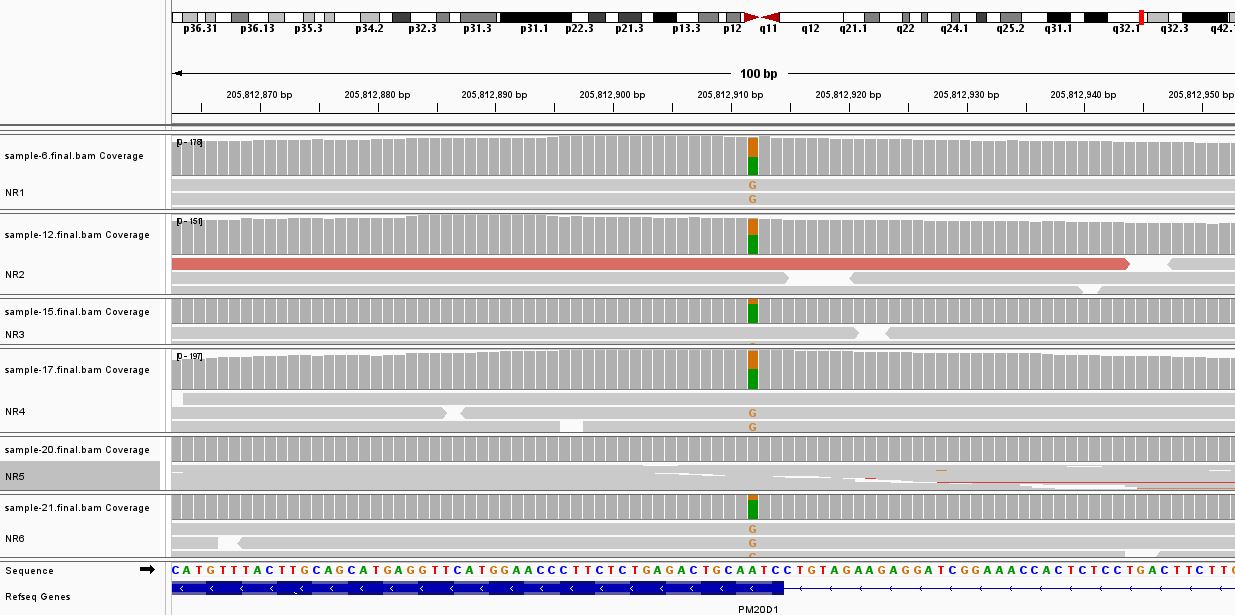 | **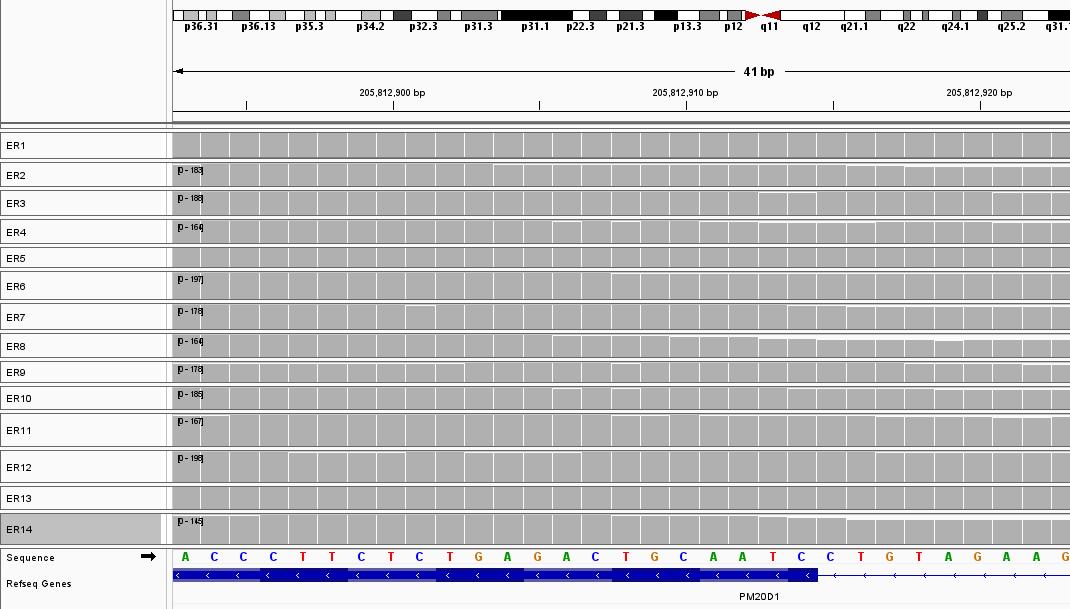** |
| CCDC18 | 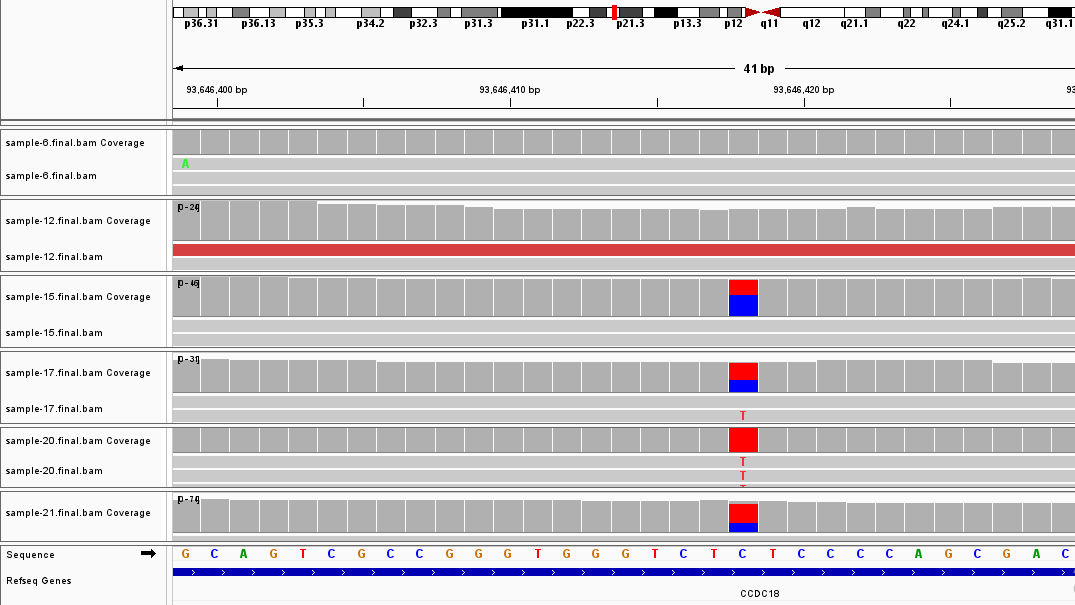 | **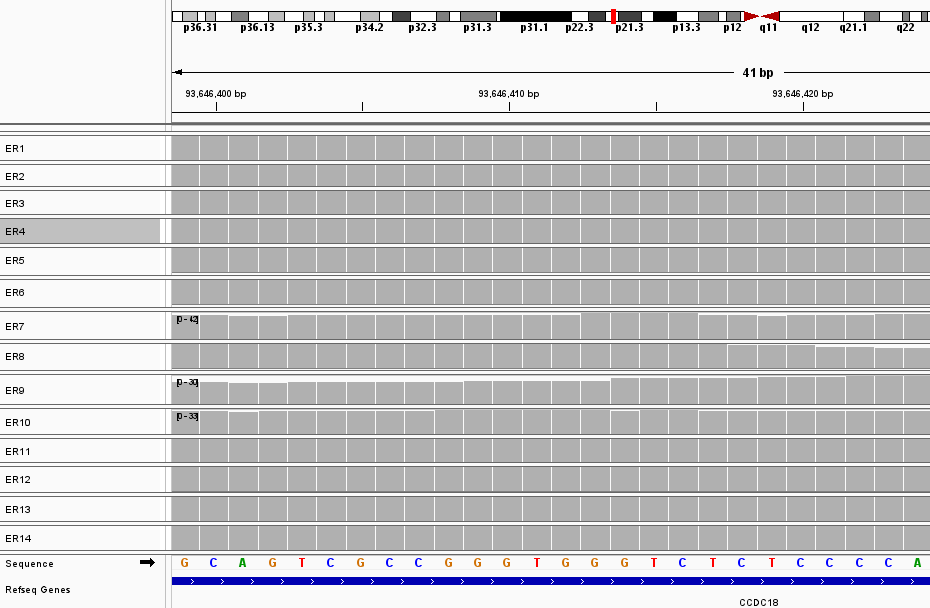** |
| LOC101929058 | 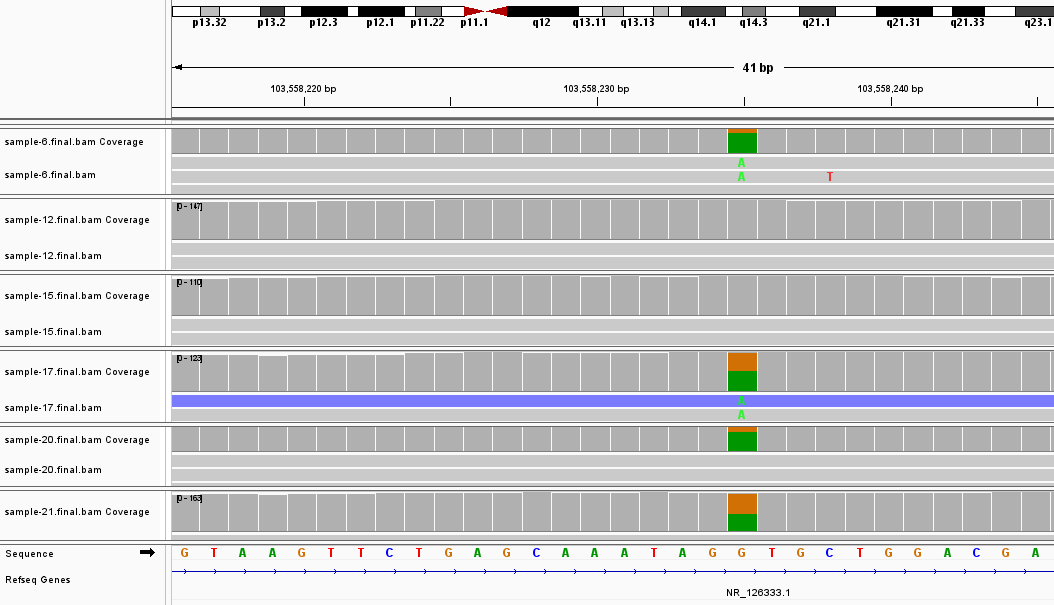 | **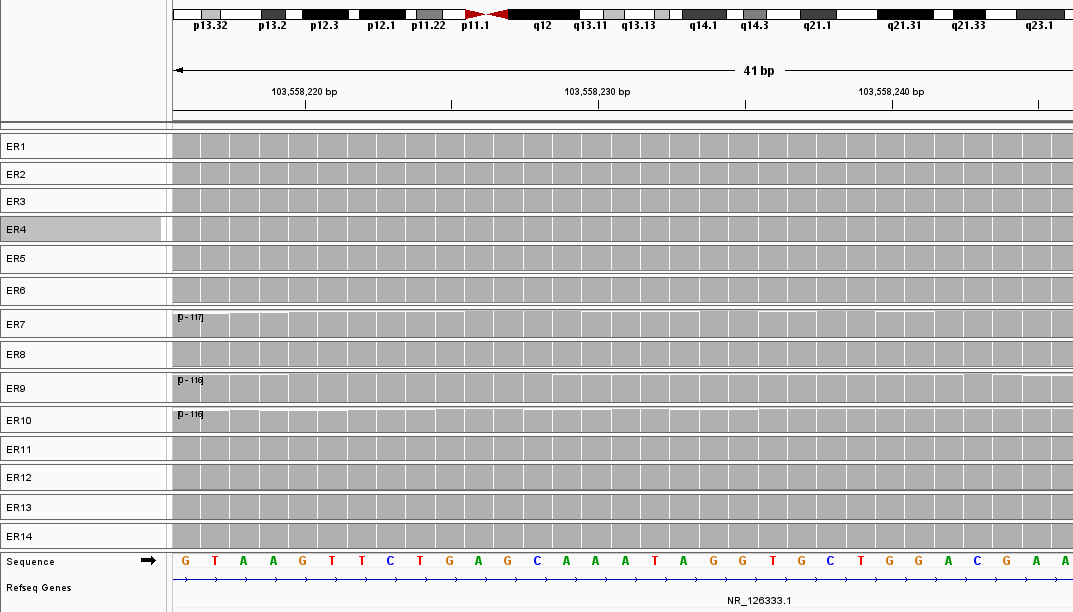** |
| CXCR4;THSD7B | 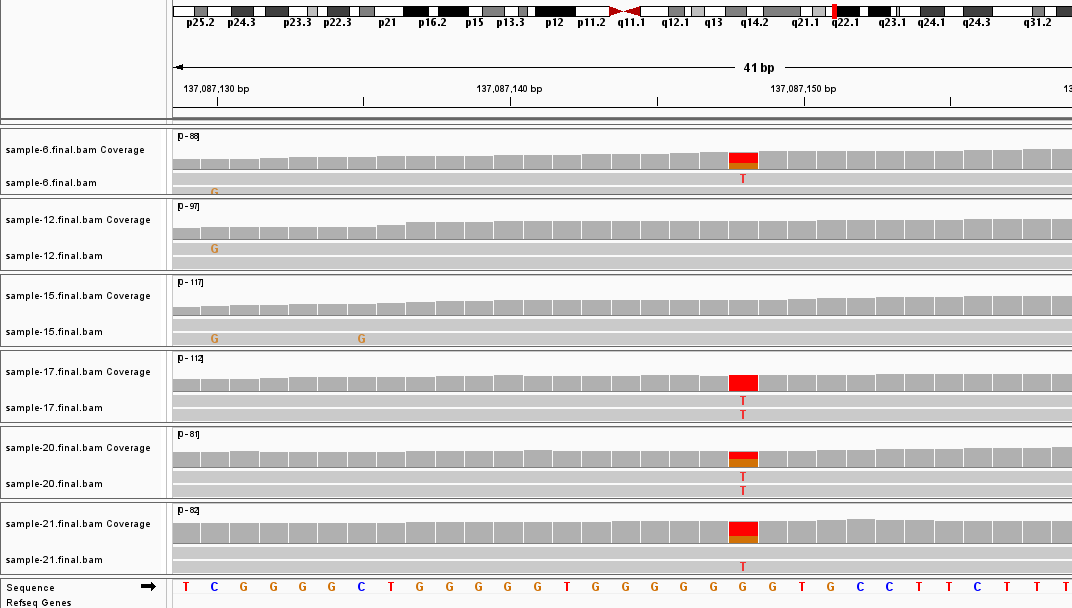 | **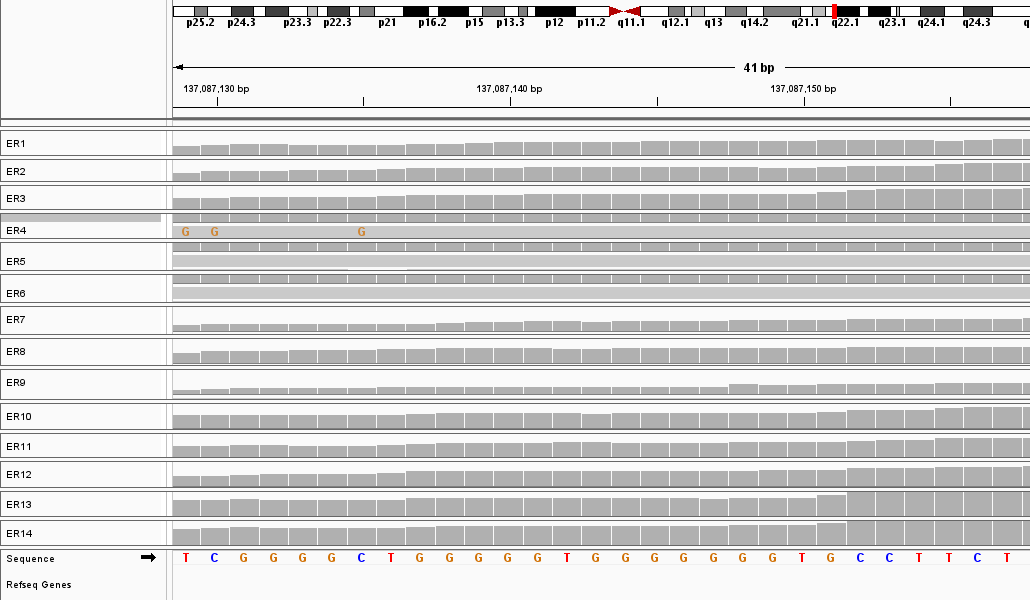** |
| SERPINA13P | 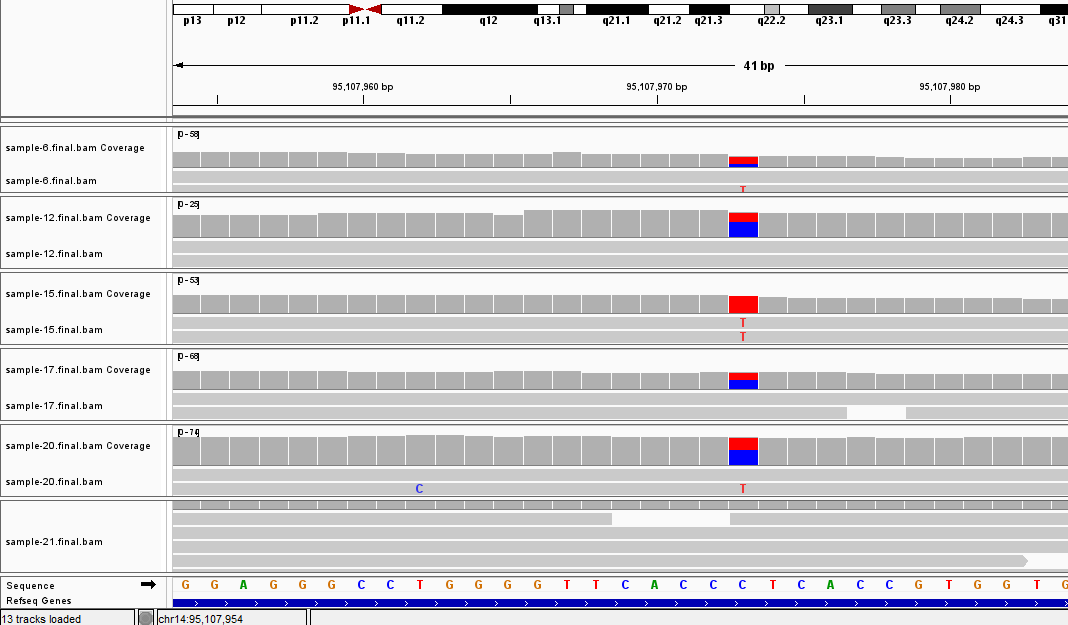 | **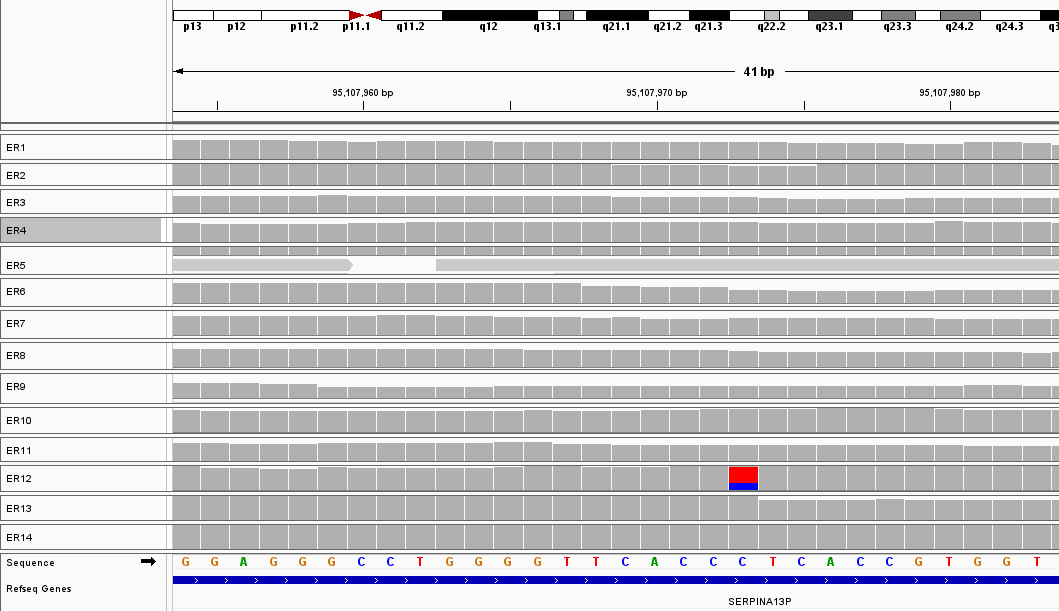** |
| SNHG14 | 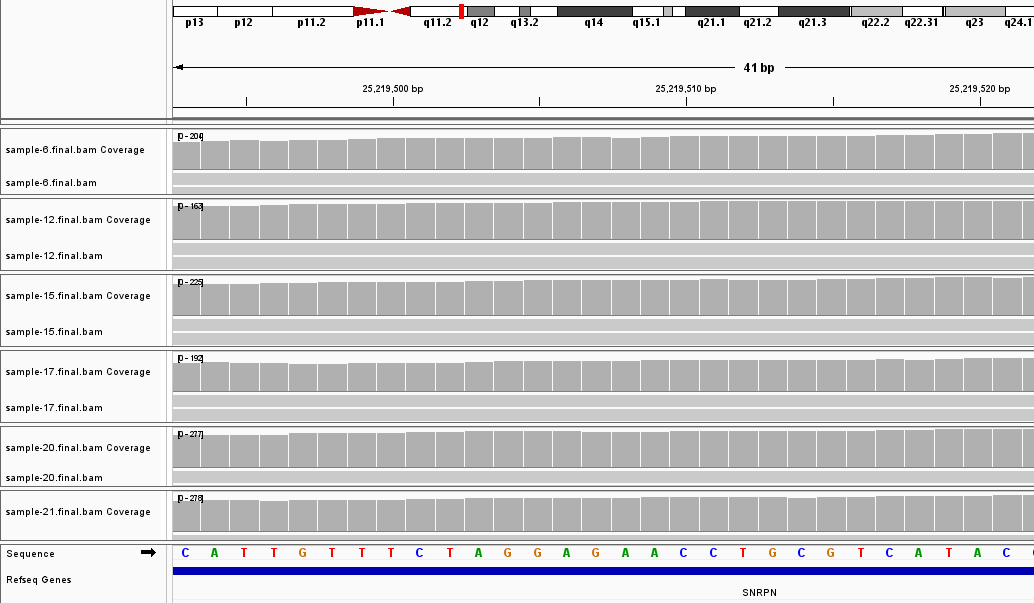 | **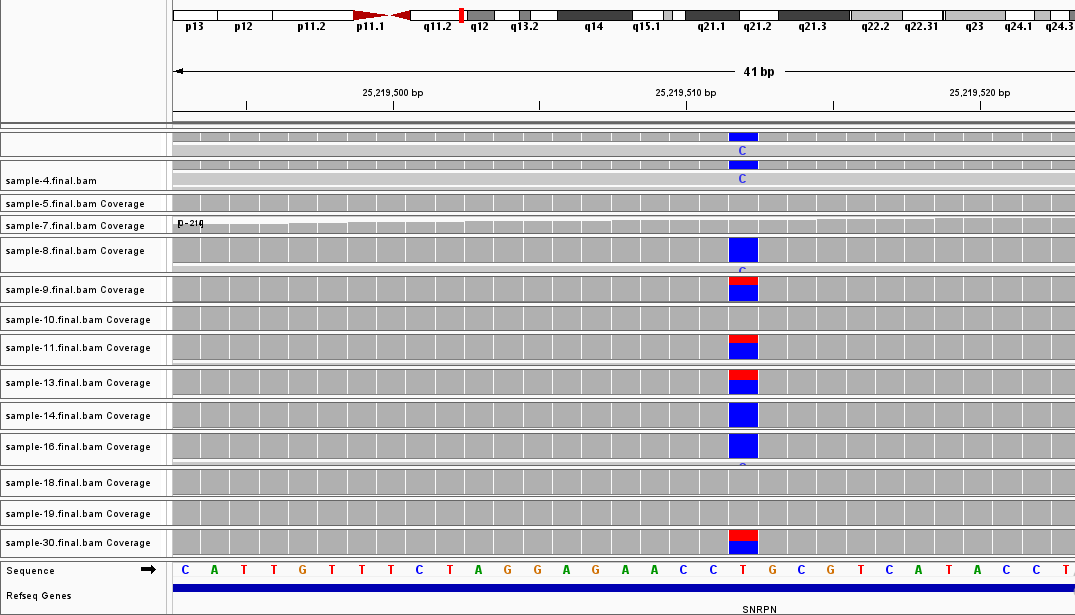** |
| NONE;CHEK2P2 | 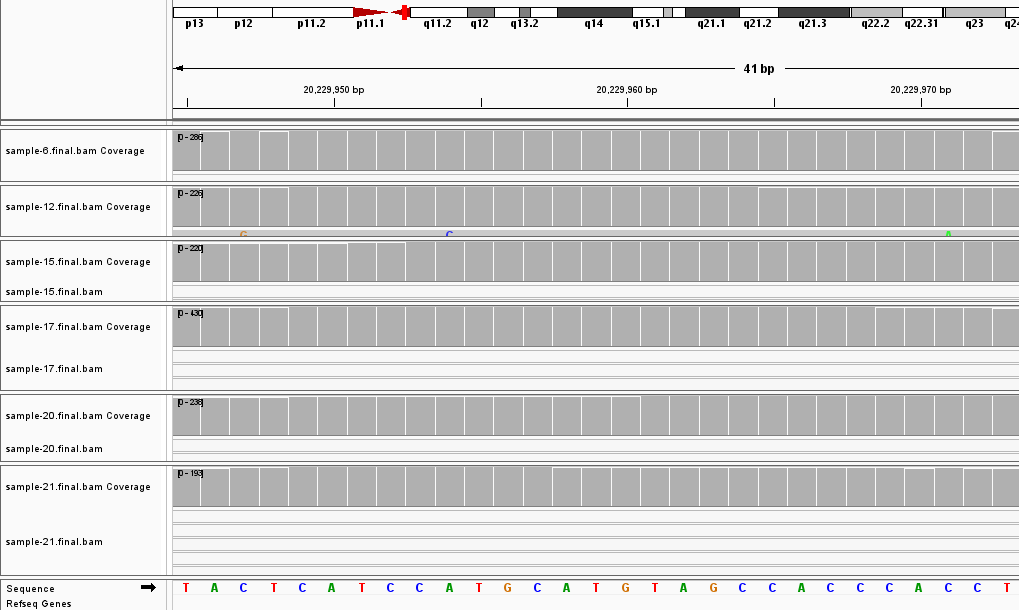 | **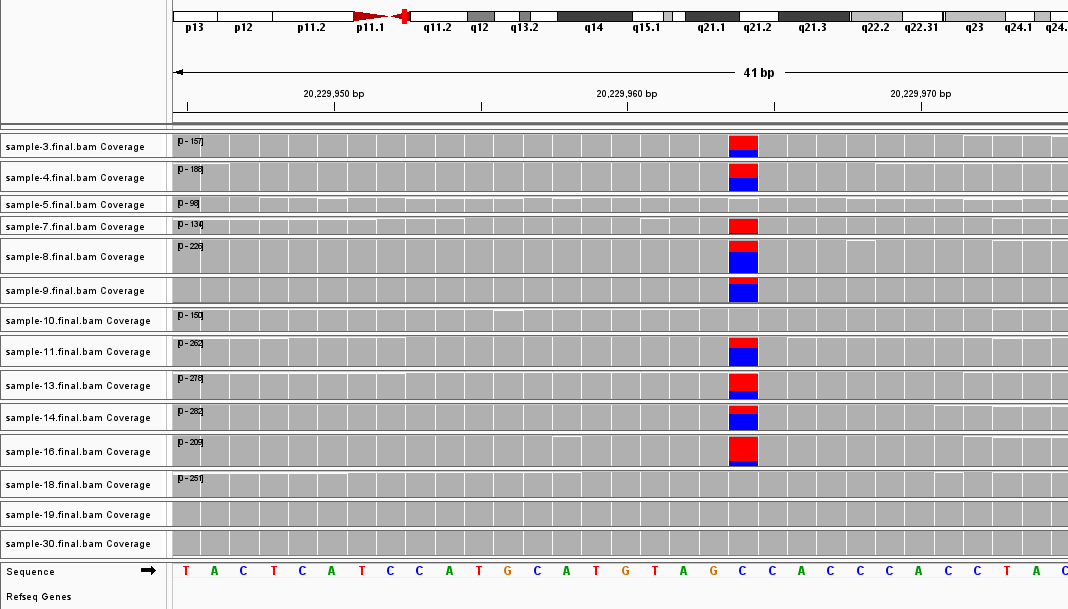** |
| ACSM2B;ACSM1 | 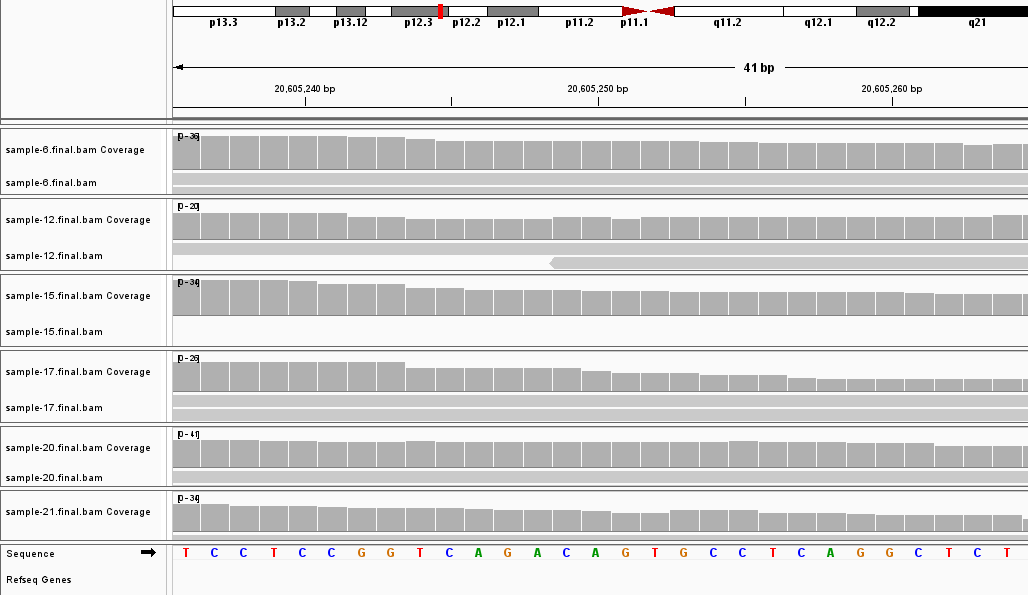 | **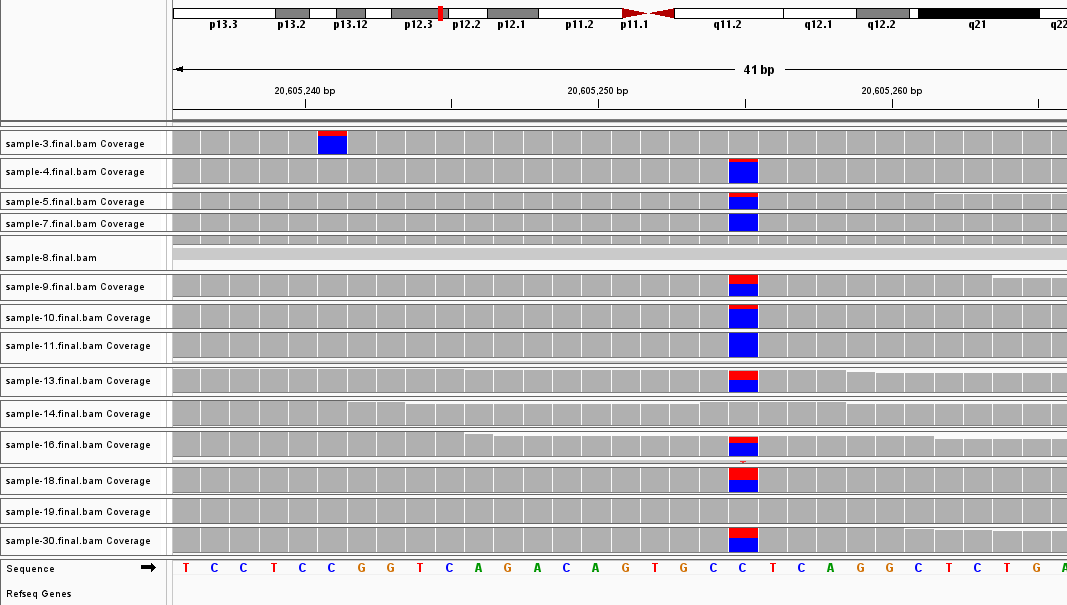** |
|  | 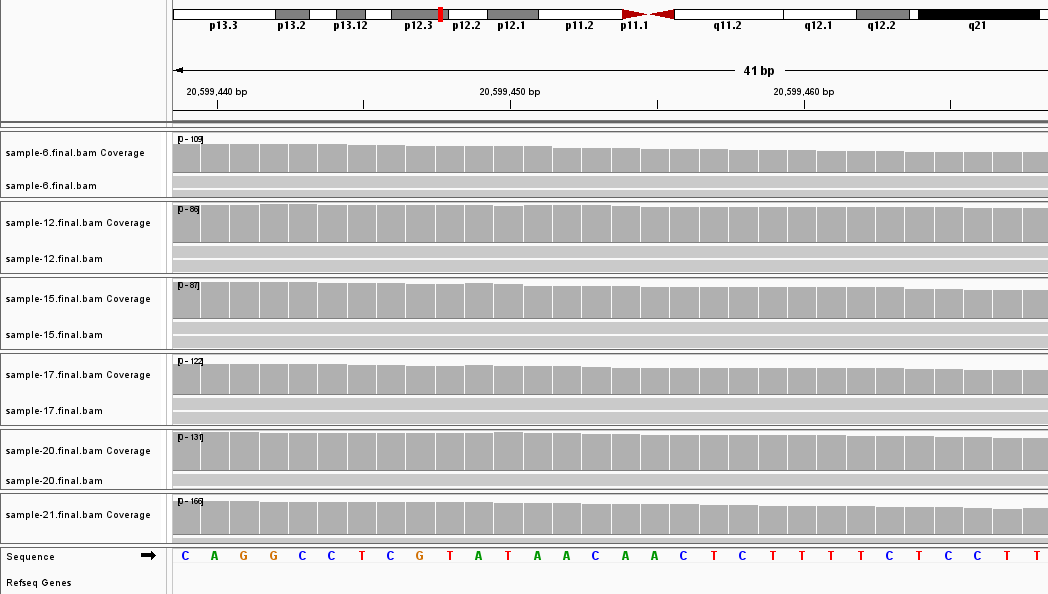 | **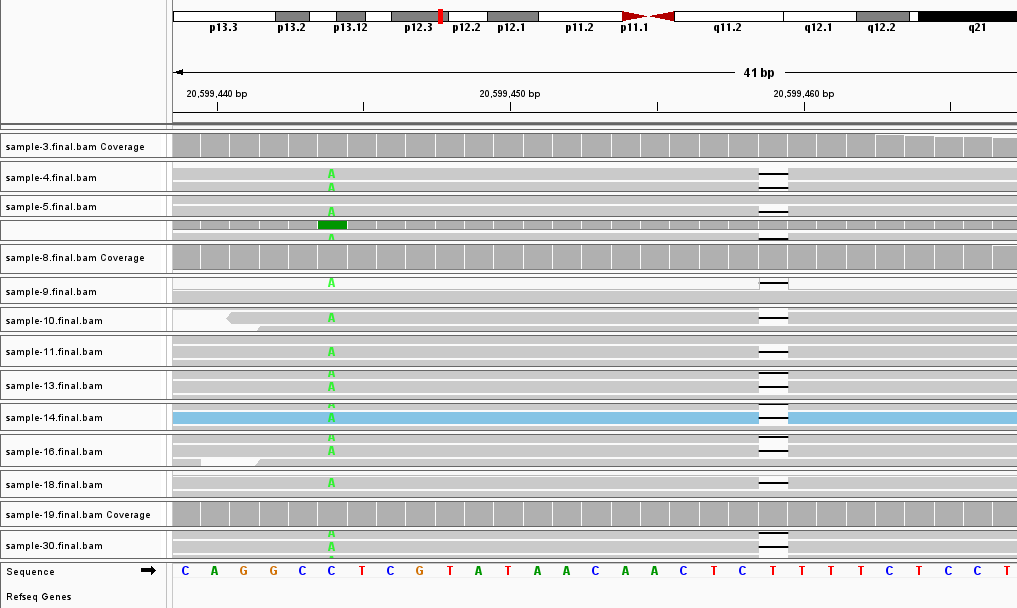** |
