# Supplementary material for: Genetic modifiers of response to thalidomide in transfusion-dependent beta-thalassemia patients: a whole-exome sequence analysis
Source: PeerJ. 2025 Oct 7;13:e20038. doi: 10.7717/peerj.20038 (PMC12513377; doi:10.7717/peerj.20038)
Supplement: Supplemental Information 6 [file peerj-13-20038-s006.docx]

| S. no | Mutated Gene symbol (as per Hugo Gene Nomenclature Committee) | Full name (as per NCBI and Ensemble recommendation) |
| --- | --- | --- |
| 1 | NPNT | Nephronectin |
| 2 | BMP3 | Bone Morphogenetic Protein 3 |
| 3 | CHD4 | Chromodomain Helicase DNA Binding Protein 4 |
| 4 | MAPK4 | Mitogen-Activated Protein Kinase 4 |
| 5 | TAF1C | TATA-Box Binding Protein Associated Factor, RNA Polymerase I Subunit C |
| 6 | INPP5D | Inositol Polyphosphate-5-Phosphatase D |
| 7 | ACP1 | Acid Phosphatase 1 |
| 8 | LGR6 | Leucine Rich Repeat Containing G Protein-Coupled Receptor 6 |
| 9 | CHI3L1 | Chitinase 3 Like 1 |
| 10 | KHNYN | KH And NYN Domain Containing |
| 11 | ZNF208 | Zinc Finger Protein 208 |
| 12 | MYH15 | Myosin Heavy Chain 15 |
| 13 | RESF1 | Retroelement Silencing Factor 1 |
| 14 | BIRC2 | Baculoviral IAP Repeat Containing 2 |
| 15 | PM20D1 | Peptidase M20 Domain Containing 1 |
| 16 | CCDC18 | Coiled-Coil Domain Containing 18 |
| 17 | *C12orf42-AS1*  (LOC101929058) | C12orf42 Antisense RNA 1 |
| 18 | CXCR4;THSD7B | C-X-C Motif Chemokine Receptor 4: Thrombospondin Type 1 Domain Containing 7B |
| 19 | SERPINA13P | Serpin Family A Member 13, Pseudogene |
| 20 | MALAT1;TALAM1 | Metastasis Associated Lung Adenocarcinoma Transcript 1: TALAM1 Transcript, MALAT1 Antisense RNA |
| 21 | SNHG14 | Small Nucleolar RNA Host Gene 14 |
| 22 | CHEK2P2 | Checkpoint Kinase 2 Pseudogene 2 |
| 23 | ACSM2B;ACSM1 | Acyl-CoA Synthetase Medium Chain Family Member 2B:  Acyl-CoA Synthetase Medium Chain Family Member 1 |
